# The importance of rating scales in measuring patient-reported outcomes

**DOI:** 10.1186/1477-7525-10-80

**Published:** 2012-07-13

**Authors:** Jyoti Khadka, Vijaya K Gothwal, Colm McAlinden, Ecosse L Lamoureux, Konrad Pesudovs

**Affiliations:** 1 NH & MRC Centre for Clinical Eye Research, Discipline of Optometry and Vision Science, Flinders Medical Centre and Flinders University of South Australia, Adelaide, South Australia, 5042, Australia; 2 Meera and L B Deshpande Centre for Sight Enhancement, Vision Rehabilitation Centres, L V Prasad Eye Institute, Hyderabad, India; 3 Centre for Eye Research Australia, Department of Ophthalmology, University of Melbourne, Victoria, 8002, Australia; 4 Singapore National Eye Centre, Singapore Eye Research Institute, Singapore, Singapore

**Keywords:** Patient reported outcomes, Rating scales, Response categories, Quality of life, Rasch analysis

## Abstract

**Background:**

A critical component that influences the measurement properties of a patient-reported outcome (PRO) instrument is the rating scale. Yet, there is a lack of general consensus regarding optimal rating scale format, including aspects of question structure, the number and the labels of response categories. This study aims to explore the characteristics of rating scales that function well and those that do not, and thereby develop guidelines for formulating rating scales.

**Methods:**

Seventeen existing PROs designed to measure vision-related quality of life dimensions were mailed for self-administration, in sets of 10, to patients who were on a waiting list for cataract extraction. These PROs included questions with ratings of difficulty, frequency, severity, and global ratings. Using Rasch analysis, performance of rating scales were assessed by examining hierarchical ordering (indicating categories are distinct from each other and follow a logical transition from lower to higher value), evenness (indicating relative utilization of categories), and range (indicating coverage of the attribute by the rating scale).

**Results:**

The rating scales with complicated question format, a large number of response categories, or unlabelled categories, tended to be dysfunctional. Rating scales with five or fewer response categories tended to be functional. Most of the rating scales measuring difficulty performed well. The rating scales measuring frequency and severity demonstrated hierarchical ordering but the categories lacked even utilization.

**Conclusion:**

Developers of PRO instruments should use a simple question format, fewer (four to five) and labelled response categories.

## Background

Patient-reported outcomes (PROs) are the measurement of patients’ perception of the impact of a disease and its treatment(s), which are typically reported via a questionnaire [1]. PROs are increasingly being accepted as the primary endpoints of clinical trials in health research [2-4]. The U.S. Food and Drug Administration (FDA) has also endorsed PROs as key clinical trial endpoints owing to the notion that such clinical trials ultimately guide patient care [5]. Therefore, it is critical that data collected by PROs are accurate and reliable, which is only possible when patients are able to understand the questions asked and select response categories that represent their status. Poorly understood questions, or underutilized rating scale categories can seriously impair the accuracy and reliability of PRO measurements [6-8].

The term rating scale generally refers to the response options that can be selected for a question or statement in a PRO instrument [7,8]. These are usually a set of categories defined by descriptive labels: rating scale categories. According to the general guidelines, rating scale categories should be presented in a clear progression (categories distinct from each other), should be conceptually exhaustive (no gaps within the range of response choices), and should be appropriate to the question of the latent trait being measured [8]. The performance of rating scale categories is also intimately connected to the format of the question [9]. Therefore, rating scale design should consider aspects of both the question format and the response categories.

The development of appropriate rating scale categories may seem straightforward; however, in the absence of high quality evidence, or consensus for the optimal methods, PRO developers take many different approaches. Perhaps the most debated issue is the optimum number of response categories. Some researchers argue that more reliable and precise measurement can be obtained with more response categories (more than seven) [10]. Whereas, others favour a small number of response categories based on the theory that fewer response options offer minimum respondent confusion and reduce respondent burden [11]. Therefore, PRO developers face a trade-off: achieve finer discrimination through a greater number of response categories versus reducing respondent burden and not exceeding the discrimination capacity of the respondents [12]. However, there are no clear guidelines available to inform this choice. Other contested issues are whether the same rating scale should be applied to all questions measuring an underlying trait, what are the optimal features involved in question formatting and what is the optimal rating scale category labelling.

In order to develop the evidence base for rating scale design, a project was undertaken to assess the rating scale used in 17 existing PRO instruments which were developed to measure the impact of cataract and/or outcomes of cataract surgery. The aim of this study was to use Rasch analysis to identify features that are characteristic of functional and dysfunctional rating scales which includes both question structure and rating scale categories across 17 PROs respectively. Our secondary aim was to develop guidelines for formulating rating scales.

## Methods

### Participants

Participants were patients on the cataract surgical waiting list at Flinders Medical Centre, Adelaide, South Australia. All participants were 18 years or older, English speaking, and cognitively able to self-administer PROs. A pack containing 10 PROs rotationally selected from the 17 PROs (Table 1) were mailed to the participants for self-administration. The study was approved by the Flinders Clinical Ethics Committee and adhered to the tenants of the Declaration of Helsinki. All participants provided written informed consent.

**Table 1:** Number of questions, rating scale used in the 17 patient reported outcomes (PROs)

**Questionnaire**	**Number of questions**	**Response options**	**Same rating scale used for all questions (Yes/No)**	**Attribute/s being assessed**
Visual Functioning index, VFI (*Bernth-Petersen, 1981*)	11	2 or 3	No	Difficulty, Severity^**†**^
Activities of Daily Vision Scale, ADVS (*Mangione* et al. *1992*)	22	5	No	Difficulty
Visual Activities Questionnaire, VAQ *(Sloane* et al. *1992)*	33	5	Yes	Frequency
Cataract Symptom Score, CSS * (Brenner* et al.*, 1993; Curbow* et al. * 1993; Javitt* et al.*, 1993)*	5	4	Yes	Severity
Visual Function Index-14, VF-14 *(Steinberg at al., 1994)*	14	5	Yes	Difficulty
Catquest (*Lundstrom* et al.*, 1997*)	24	2^**‡**^ or 4	No	Difficulty, Frequency, Severity
Visual Function and Quality of Life, VF&QOL (*Fletcher* et al.*, 1997*)	25	4	No	Difficulty, Global rating of vision
Quality of Life and Visual Function, QOLVFQ (*Carta at al., 1998***)**	17	3	Yes	Difficulty
Visual Disability Assessment, VDA (*Pesudovs and Coster, 1998*)	18	4	Yes	Difficulty
Vision Core Measure 1, VCM1 (*Frost* et al.*, 1998*)	10	6	Yes	Frequency
Cataract Symptom Scale, CSScale (*Crabtree* et al.*, 1999*)	15	5	No	Difficulty, Frequency
Impact of Cataract Surgery, ICS (*Monestam and Wachtmeister, 1999*)	4	4	No	Difficulty^**+**^
Technology of Patient Experiences, TyPE (*Javitt* et al.*, 1999*)	13^*^	5	No	Global rating of vision, Difficulty
Houston Vision Assessment Test, HVAT (*Prager at al., 2000*)	10	5^**ξ**^	No	Difficulty, Severity
Impact of Vision Impairment, IVI (*Hassell* et al.*, 2000*)	32	6	No	Difficulty/Severity, Frequency
National Eye Institute-Visual Function Questionnaire, NEIVFQ *(Mangione* et al.*, 2000)*	39^**#**^	5, 6 or 11^**^**^	No	Difficulty, Global rating of health, Global rating of vision, Frequency, Severity
Visual Symptoms and Quality of Life, VSQ *(Donovan* et al.*, 2003)*	26	8, 7, 5, 4, 3 or 2^**$**^	No	Difficulty, Frequency, Global rating of vision

^**†**^one more attribute assessed was descriptive and could not be classified.

^**‡**^ questions that used 2 response categories are related to demographics and are not used in calculation of score.

^*^ there are additional questions related to demographics which are not included in the calculation of the overall score.

^**†**^ one more attribute assessed was descriptive and could not be classified.

^**ξ**^ each question has two parts and the categories are multiplied to obtain the total score for a question. Thus, there are 10 categories as a result of multiplicative categories.

^**#**^ A shorter version of NEIVFQ with 25 questions is also available.

^**^**^ response category option for question number 11 was unlabelled.

^**$**^ response categories varied depending on the question.

### Questionnaires

A systematic literature search was performed for PROs that were used to measure the impact of cataract and/or outcomes of cataract surgery on a polytomous rating scale (rating scale with more than 2 response categories) in Entrez PubMed. Seventeen PROs met the criteria (Table 1). The 17 PROs (items listed in Additional file 1: Appendix) assess various vision-related quality of life dimensions using ratings of the following four concepts:

• Difficulty: e.g. “*Do you have difficulty reading small print*?” (No difficulty at all = 0, A little difficulty = 1, Moderate difficulty = 2, Very difficult = 3, Unable to do = 4, Don’t do for reasons other than sight/not applicable = 5).

• Frequency: e.g. *“In the past month, how often have you worried about your eyesight getting worse?”* (Not at all = 0, Very rarely = 1, A little of the time = 2, A fair amount of the time = 3, A lot of the time = 4, All the time = 5)

• Severity: e.g. “*How much pain or discomfort have you had in and around your eyes?”* (None = 1, Mild = 2, Moderate = 3, Severe = 4, Very severe = 5).

• Global ratings: e.g. “*In general would you say your vision (with glasses, if you wear them) is*…” (Very good = 1, Good = 2, Fair = 3, Poor = 4).

### Rasch analysis

Rasch analysis is a probabilistic mathematical model that estimates interval-scaled measures from ordinal raw data [13]. Rasch analysis also provides a strong assessment of rating scale functioning. Interested readers are directed to the article by Mallinson for further information on ordinal versus interval data [14], a chapter by Hays for a non-technical description of Rasch models [15] and the paper by Linacre on rating scale category analysis [8].

### Assessment of the rating scale

**Figure 1:**
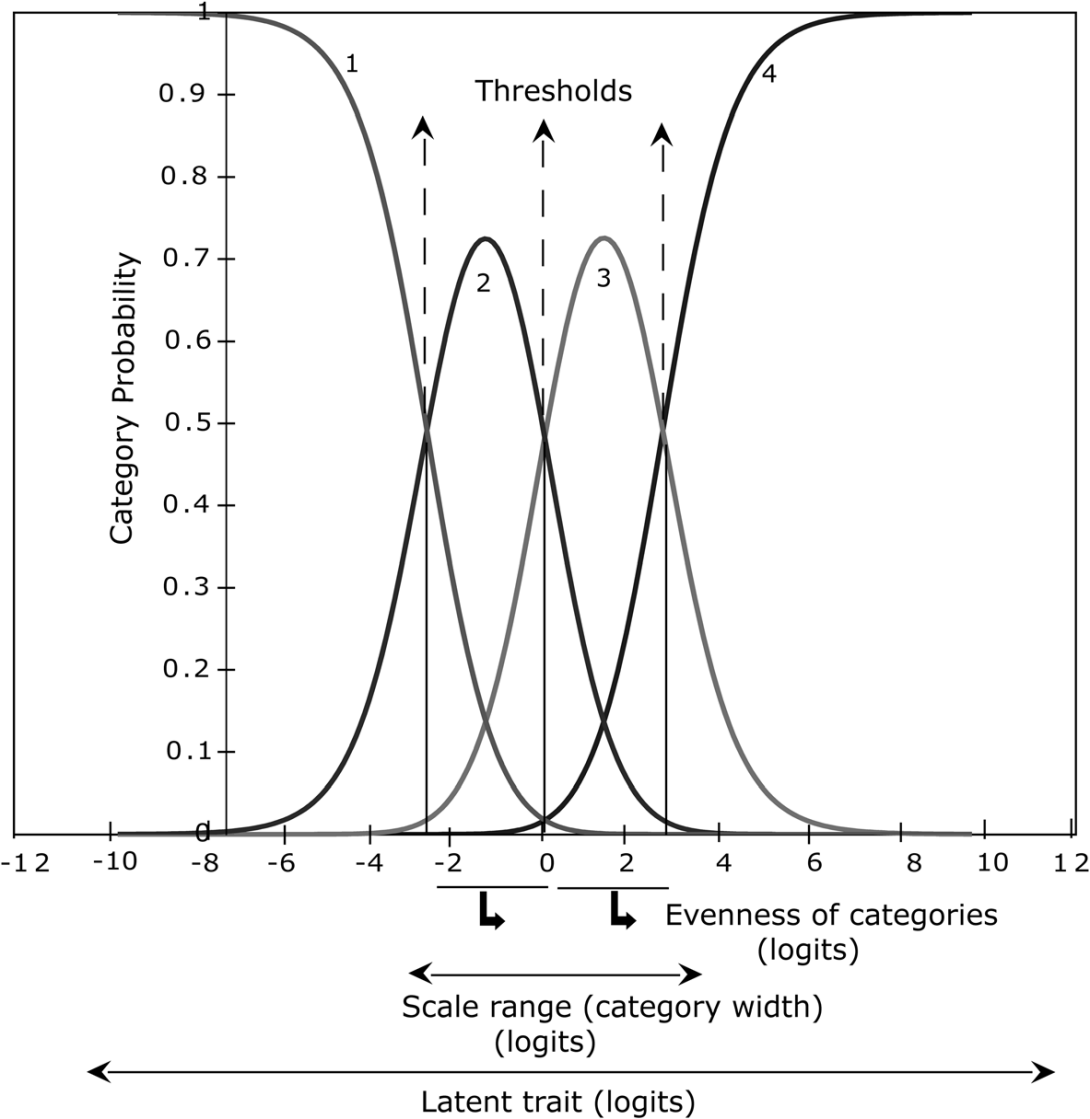
**Rasch model category probability curves of a question with four response categories (1, not at all; 2, a little; 3, quite a bit; and 4, a lot).** The *x*-axis represents the attribute in logits. The *y*-axis represents the probability of a response category being selected. The curves represent the likelihood that a respondent with a particular amount of the latent trait will select a category: illustration of the concepts of scale range (−3 to +3, i.e. 6 logits in this example), 3 thresholds for 4 categories and evenness of categories (category width, 3 logits each; standard deviation of the width, 0).

**Figure 2:**
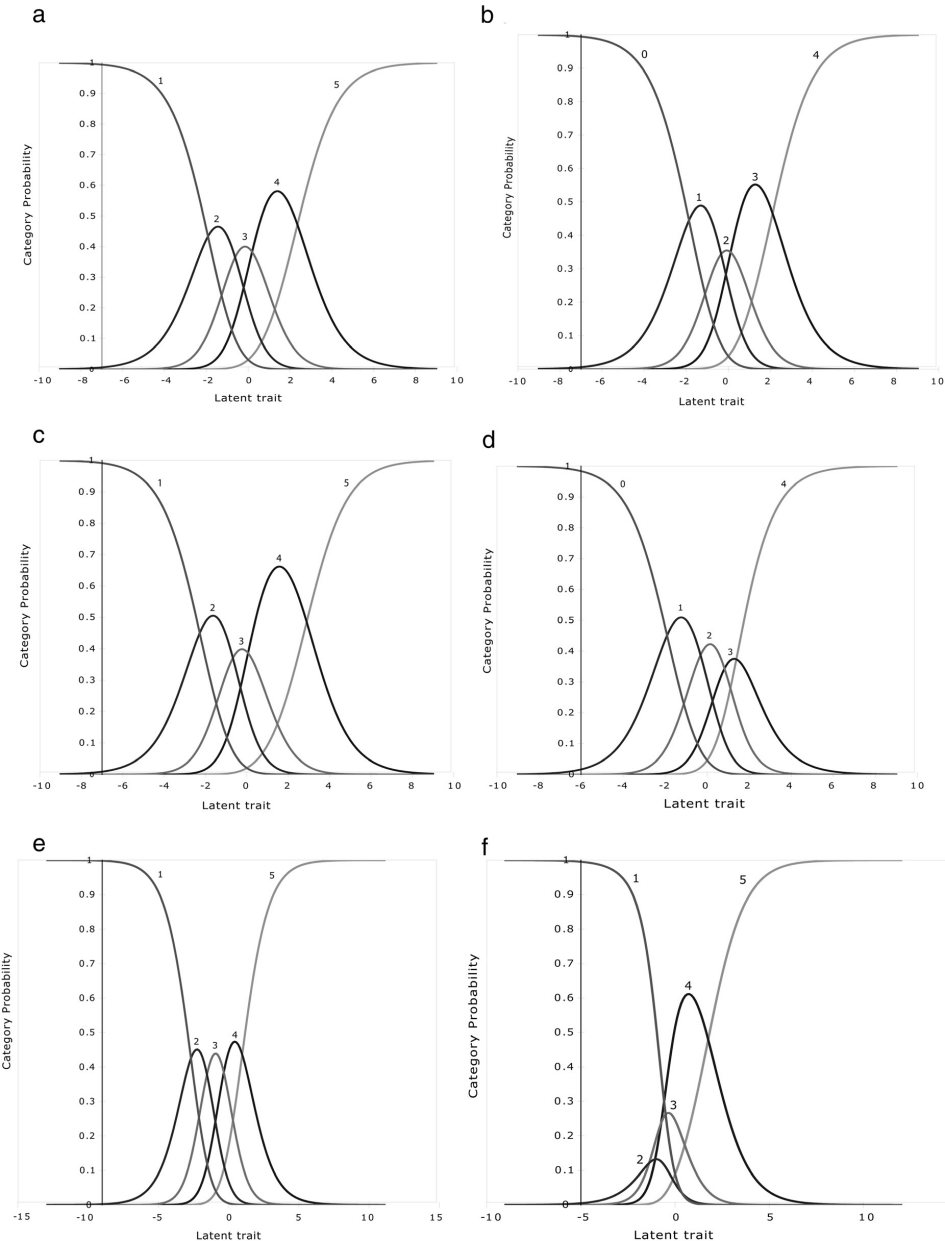
**a–e Rasch model category probability curves showing functional rating scales for items with five response categories that assess ‘difficulty’ in five different questionnaires: (a) **Visual Symptoms and Quality of Life Questionnaire, VSQ (Question numbers 1, 6, 8 and 9). Response categories of 1–5 correspond to ‘no difficulty’, ‘yes, a little difficulty’, ‘yes, some difficulty’, ‘yes, a great deal of difficulty’ and ‘I cannot perform the activity because of my eyesight’. (**b**) Cataract Symptom Scale, CSS (all). Response categories of 0–4 correspond to ‘no’, ‘a little difficulty’, ‘a moderate difficulty’, ‘very difficult’ and ‘unable to do’. (**c**) Technology of Patient Experiences (Question numbers 2–13). Response categories of 1–5 correspond to ‘not at all’, ‘a little bit’, ‘some’, ‘quite a lot’, and ‘totally disabled’. (**d**) Visual Function-14, VF-14 (all). Response categories of 0–4 include ‘unable to do the activity’, ‘a great deal, ‘a moderate amount’, ‘a little’ and ‘no’. (**e**) National Eye Institute –Visual Function Questionnaire NEIVFQ (Question numbers 5–16). Response categories of 1–5 include ‘no difficulty at all’, ‘a little difficulty’, ‘moderate difficulty’, ‘extreme difficulty’ and ‘stopped doing this because of eyesight’. Figure 2 (**f**) Rasch model category probability curves showing disordered thresholds for five- response category questions that assess ‘difficulty’ in Activities of Daily Living Scale (ADVS). The peak of the two middle categories 2 and 3 are submerged and the thresholds are disordered which represents that the respondents had difficulty discriminating adjacent categories.

Rating scale functioning can be assessed visually on a category probability curve graph (CPC) which displays the likelihood of each category being selected over the range of measurement of an underlying trait (Figure 1). Each curve in the CPC represents a response category. An important landmark in the CPC is the “threshold”. The threshold is the point at which two neighbouring response categories intersect (Figure 1). At this intersection, a respondent has equal likelihood of choosing one category or the other [16]. The number of thresholds is always one less than the number of response categories, so there are three thresholds for a four-response category. In the well-functioning rating scale shown in Figure 1, thresholds are arranged in a hierarchical order, which is demonstrated by each curve showing a distinct peak, illustrating the position along the continuum (linear scale) where the categories are most likely to be selected [17,18]. The distance between two neighbouring thresholds defines the size of intervening category. Figure 2a - 2e demonstrate ordered category thresholds, suggesting that the respondents were able to discriminate between these response categories. However, thresholds may not always show ordered arrangement which indicates that the respondents have either not been able to use all categories or had difficulty discriminating between response categories (Figure 2f) [19,20]. Such rating scales are dysfunctional and require modifications. For this study, we used the following three criteria to evaluate functioning of the rating scales:

1. **Ordered thresholds:** This is the fundamental characteristic of a rating scale. Failing to demonstrate ordered thresholds indicates that the choices in the rating scale do not follow the expected hierarchical ordering. Such a rating scale is dysfunctional. Other characteristics (evenness of categories and scale range) are inconsequential when the rating scale has disordered thresholds. Therefore, if the rating has disordered thresholds then the other two criteria were not evaluated.

2. **Evenness of categories:** This indicates the relative utilization of response categories by the respondents. It is represented by the standard deviation (SD) of the category widths; the smaller the SD, the more even the categories widths. On the contrary, a dysfunctional rating scale can have categories too close together (indicating overlapping categories) or too far apart from each other (indicating need for more categories).

3. **Scale range:** Scale range is the distance between the first and the last category threshold in a rating scale. This indicates the spread of the response categories on the scale range (Figure 1). Larger scale ranges result in greater measurement coverage of the latent trait.

The fit statistics of all the items were also assessed. The fit statistics indicate how well items fit to the Rasch model. There are two types of fit statistics; infit and outfit. Both types of the fit statistics are measured as mean square standardized residuals (MNSQ). The expected statistic is 1.0, with the deviation from this value indicating under- or over-fit. A strict range for acceptable MNSQ is from 0.7 to 1.3, however, a more lenient range of 0.5 and 1.5 is considered productive for the measurement [21,22]. In this paper, we have considered the lenient range (MNSQ, 05–1.5) as fit to the Rasch model.

This study aims to report the characteristics of rating scale categories in their original format for all items across the 17 different PRO instruments. Interested readers are requested to refer to a series of publications by our group which report how the Rasch analysis was used to optimize the other aspects of measurement properties of these 17 PRO instruments [23-38].

### Statistical analysis

Rasch analysis was performed for qualitative and quantitative assessment of the rating scale with Winsteps software (version 3·68) using the Andrich rating scale model for polytomous data [18,39].

## Results

Six hundred and fourteen patients completed at least one PRO instrument. The average response rate for the 17 PRO instruments was 45%. The mean age of the participants was 74·1 years (SD, ± 9.4) and 56% were female. Among the 614 patients, 59% had bilateral cataract, 41% were awaiting second eye surgery and 51% had ocular co-morbidities (glaucoma, 16%; age-related macular degeneration, 9%; and diabetic retinopathy, 4%). The participants had been diagnosed with cataract for an average of 3.2 ± 8.7 years. The mean visual acuity was 0.22 ± 0.20 LogMAR (~6/9.5^−1^) in the better eyes and 0.55 ± 0.36 LogMAR (~6/24^+2^) in the worse eyes. Participants had systemic co-morbidities representative of the elderly cataract population in Australia [40].

### Dysfunctional rating scales

Dysfunctional rating scales were found in five of the 17 PROs and were observed for ‘difficulty’ and ‘global vision’ but not for frequency and severity ratings. The PROs with a large number of response categories showed greater numbers of disordered thresholds (Table 2). Disordered thresholds were also evident for PROs with a complicated question layout such as the Activities of Daily Living Scale (ADVS). In the ADVS, items are branched into two-parts (e.g. item 1a: *“would you say driving at night with”,* item 1b: *“is it because of your visual problems that you are unable to drive at night?”*). Similarly, the PROs with conceptually similar category labels (e.g. Impact of Visual Impairment [IVI]: “*not at all, hardly at all, a little, a fair amount, a lot and can’t do because of eyesight”*) and unlabelled categories (e.g. ten-category global rating scale of the National Eye Institute Visual Function Questionnaire, [NEIVFQ]) also demonstrated disordered thresholds.

**Table 2:** Dysfunctional rating scales

**Attribute under assessment**	**Questionnaire (Question number)**	**Number of categories**	**Categories labelled**	**Number of ordered thresholds/Total number of thresholds**	**Misfitting items/ Total items**	**Reasons for dysfunction**
**Difficulty**						
	IVI (1–19)	6	✓	3/5	1/19	Poorly defined categories (overlapping categories)
	ADVS (1bc–19 bc except 6 and 7bc)	5	✓	3/4	6/17	Question format (complicated layout due to branching question)
**Others**						
Limitation	HVAT (1ab-10ab)	10	X	6/10	2/10	Too many categories (multiplicative scale)
Global rating (Health)	NEIVFQ (A1, A2)	10	X	8/9	0/2	Too many categories and unlabelled
True/False	NEIVFQ (20–25, A12, A13)	5	✓	2/4	6/8	Use of neutral (‘unsure’) middle category
Apprehension	ADVS (6bc, 7bc)	5	✓	3/4	0/2	Complicated question format (complicated lay out due to branching question)
Descriptive	ICS (1)	4 (3^rd^ category not used)	✓	0/2	1/1	Complicated question format (complicated lay out due to branching question)
	VSQ (16)	3	✓	1/2	1/1	Complicated question format (very long description of categories)

For non-abbreviated name of the PROs, please refer Table 1.

Rating scale labels for above PROs are:

Difficulty

IVI (1–19)- Not at all, hardly at all, a little, a fair amount, a lot, cannot do because of eyesight; ADVS (1bc-19bc, except 6bc and 7bc)–No difficulty at all, a little difficulty, moderate difficulty, extreme difficulty, unable–yes (go to next question), no (go to next question).

Others

HVAT (1A-10B)- Part A: Not at all limited, slightly limited, somewhat limited, moderately limited, severely limited; Part B- I have no visual or physical limitations; none due to eyesight, some due to eyesight, half due to eyesight, most due to eyesight/all due to eyesight; NEIVFQ(20–25,A12,A13)- Definitely true, mostly true, not sure, mostly false, definitely false; NEIVFQ(A1,A2)- Worst to best (unlabelled); VSQ (16)- No, I have not had to give up work, I have not had to give up work but I am having difficulty because of my eyesight, I am retired/gave up work because of trouble with my eyesight; ADVS (6bc, 7bc)- No apprehension at all, a little apprehension, moderate apprehension, extreme apprehension, unable–yes (go to next question), no (go to next question); ICS (1)- Yes, No, If yes–none, spectacles, hand, stand magnifiers, others.

### Functional rating scales

The characteristics of rating scales that demonstrated functional response options are shown in Table 3 (difficulty and frequency) and Table 4 (severity and global vision). Similarly, Figure 2 (a–e) illustrate the performance of difficulty rating scales from five PROs (Visual Symptoms and Quality of life [VSQ]; Cataract Symptom Score [CSS]; Technology of Patient Experience [TyPE]; Visual Function Index [VF14]; and NEIVFQ).

**Table 3:** Functional rating scales addressing difficulty and frequency attributes

**Attribute under assessment**	**Questionnaire (Question number)**	**Number of categories**	**Categories labelled**	**Ordered thresholds/ Number of thresholds**	**Scale range (logits)**	**Width of each category (logits)**	**Standard deviation**	**Misfitting items/ Total items**
**Difficulty**								
	VSQ (1,6,8,9)	5	✓	4/4	6.50	2.56, 1.13, 2.81	0.91	0/4
	CSScale (1–10)	5	✓	4/4	3.97	1.53, 0.45, 1.99	0.79	0/10
	TyPE (2–13)	5	✓	4/4	3.67	1.03, 0.36, 2.28	0.97	1/18
	NEIVFQ (5–16, A3-A9)	5	✓	4/4	3.65	1.14, 1.20, 1.31	0.09	1/19
	VF-14 (1–12)	5	✓	4/4	7.22	3.91, 2.12, 1.19	1.38	4/12
	VSQ (3,5,7,VS4)	5	✓	4/4	4.59	2.77, 1.63, 0.19	1.29	0/4
	VF&QOL (2–15c)	4	✓	3/3	5.04	2.67, 2.37	0.21	0/2
	VDA (1–18)	4	✓	3/3	4.05	2.22, 1.83	0.28	0/18
	Catquest (7–14, 16,17, 24)	4	✓	3/3	2.90	2.54, 0.36	1.54	0/11
	QOLVFQ (1–17)	3	✓	2/2	3.54	3.54	**–**	0/17
	ICS (3)	3	✓	2/2	2.46	2.46	–	0/1
**Frequency**								
	IVI (20–32)	6	✓	5/5	3.68	0.12, 1.76, 0.58, 1.22	0.72	0/12
	NEIVFQ (3,17–19, A11a and b)	5	✓	4/4	3.58	0.69, 1.76, 1.13	0.54	1/6
	VAQ (1–33)	5	✓	4/4	3.55	0.60, 0.09, 0.98	0.45	1/33
	CSScale (11–15)	5	✓	4/4	2.93	1.01, 0.58, 1.34	0.38	1/5
	VSQ (4,10–14, VS5)	4	✓	3/3	3.53	3.07, 0.46	1.85	0/6
	VSQ (VS1)	4	✓	3/3	3.85	2.51, 1.34	0.83	0/1
	Catquest (1–6, 23)	3	✓	2/2	2.78	2.78	–	4/7

For non-abbreviated name of the PROs, please refer Table 1.

Rating scale labels for above PROs are:

Difficulty

VSQ (1, 6, 8, 9)-No difficulty/yes, a little difficulty/yes, some difficulty/yes, a great deal of difficulty/yes, I cannot perform the activity because of my eyesight; Cataract Symptom Scale (1–10)- No, a little difficulty, a moderate difficulty, very difficult, unable to do; TyPE (2–13)-Not at all, a little bit, some, quite a lot, totally disabled; NEI VFQ (5–16, A3–A9)- No difficulty at all, a little difficulty, moderate difficulty, extreme difficulty, stopped doing this because of eyesight; VF-14 (all)–Yes,No,Not applicable, If yes–a little, a moderate amount, a great deal, unable to do the activity; VSQ (3, 5, 7, VS4)- No, not difficult/yes, a little difficult/yes, quite difficult/yes, very difficult/I cannot perform the activity; VSQ (VS8)-Not at all/a little/quite a lot/a great deal; VF&QOL (2–15c)–Not at all/a little/quite a lot/a lot; VDA (all)–Not at all/a little/quite a bit/a lot; Catquest (7–14, 16, 17, 24)–Yes, extreme difficulty/yes, much difficulty/yes, some difficulty/no, no difficulty; QOLVFQ (1–17)–Not at all, quite a lot, very much; ICS (3)- No problems, some problems, severe problems.

Frequency

IVI (20–32)- Not at all, very rarely, a little of the time, a fair amount of the time, a lot of the time, all the time; NEIVFQ (3, 17–19, A11a, A11b)-None of the time, a little of the time, some of the time, most of the time, all of the time; VAQ (1–33)- Never, rarely, sometimes, often, always; Cataract Symptom Scale (11–15)–No, occasionally, sometimes, most of the time, all of the time; VSQ (4, 10–14, VS5)–No, never/yes, some of the time/yes, most of the time/yes, all of the time; VSQ (VS1)–No, occasionally, sometimes, most of the time, all of the time; Catquest (1–6, 23)–No, Yes/If yes, once a week, at the most 2–4 times a week (approx.), daily.

**Table 4:** Functional rating scales addressing severity and global attributes

**Attribute under assessment**	**Questionnaire (Question number)**	**Number of categories**	**Categories labelled**	**Number of ordered thresholds/ Total number of thresholds**	**Scale range (logits)**	**Width of each category (in logits)**	**Standard deviation**	**Misfitting items/Total items**
**Severity**								
	NEIVFQ (4)	5	✓	4/4	4.14	2.17, 1.43, 0.54	0.82	0/1
	Catquest (15)	4	✓	3/3	4.65	2.07, 2.53	0.36	0/1
	CSS (all questions)	4	✓	3/3	2.89	1.50, 1.39	0.08	0/1
	VFI (2)	3	✓	2/2	3.60	3.60	–	0/1
**Global**								
Global rating of vision	VSQ (V2, V3)	8	✓	7/7	7.04	2.11, 0.8, 0.82, 0.93, 1.00, 1.38	0.50	2/2
Global rating of vision	VSQ (V1)	7 (7^th^ category not used)	✓	5/5	9.11	3.09, 1.44, 1.90, 2.68	0.75	0/1
Global rating	VSQ (VS6)	7	✓	6/6	5.28	0.70, 0.26, 1.24, 0.36, 2.72	1.01	0/1
Global rating of vision	NEIVFQ (2)	6	✓	5/5	10.18	3.23, 2.44, 1.38, 3.18	0.86	0/1
Global rating (health)	NEIVFQ (1)	5	✓	4/4	5.70	1.59, 1.89, 2.22	0.32	0/1
Global rating of vision	TyPE (1)	5 (5^th^ category was not used)	✓	3/3	3.89	2.62, 1.27	0.95	0/1
Global rating/ severity	VF&QOL (1)	4	✓	3/3	6.20	3.36, 2.84	0.37	0/1
	VFI (1)	3	✓	2/2	6.08	6.08	–	0/1

For non-abbreviated name of the PROs, please refer Table 1.

Rating scale labels for above PROs are:

Severity

NEIVFQ(4)-None, mild, moderate, severe, very severe; Catquest (15)- Very dissatisfied, rather dissatisfied, rather satisfied, very satisfied; Cataract Symptom Score (1–5a)–Yes, No/If so–very bothered, somewhat bothered, a little bothered; VF1 (2)**-** Good, moderate, poor.

Global

VSQ (V2, V3)–Excellent, very good, quite good, average, quite poor, very poor, appalling, cannot see at all; VSQ (1)–Excellent, very good, quite good, average, quite poor, very poor, appalling; VSQ (VS6)–Perfectly happy, pleased, mostly satisfied, mixed feelings, mostly dissatisfied, very happy, desperate; NEIVFQ (2)–Excellent, good, fair, poor, very poor, completely blind; NEIVFQ (1)- Excellent, very good, good, fair, poor; TyPE (1)- Poor, fair, good, very good, excellent; VFQOL (1)**–**Very good, good, fair, poor; VFI (1)- Nothing, large printing types, small printing types.

### Difficulty ratings

The number of categories with ‘difficulty’ questions ranged from three to five. There were 13 different rating scale formats used in “difficulty” questions, six of which were anchored with “No difficulty” or “Not at all” at one end, and “Unable to do” or “Stopped doing because of eyesight” at the other. In the majority, the first response category represented the most positive option (i.e. “No difficulty”).

Across PROs, there were six different formats of “difficulty” questions with five response categories (Table 3). There was a large variation in the scale range of these categories (2·46 to 7.22 logits). With a simple question format (e.g. item 1: *“do you have difficulty recognising people’s faces because of trouble with your eyesight?”*) and the five-category option, the VSQ demonstrated a large scale range (6.50 logits), however, response categories showed some unevenness (high SD, 0·91). With a narrower scale range (4.05 logits), the Visual Disability Assessment (VDA) was the best performing PRO with four-response categories in terms of evenness of categories (small SD, 0·28). The VDA follows a simple and uniform question format (e.g. item 4: *To what extent, if at all, does your vision interfere with your ability to watch TV?*) and categories *(“not at all, a little, quite a bit and a lot*”) across all the items. For difficulty rating, increasing the number of categories did not always provide a larger coverage of the latent trait and often introduced unevenness of the categories (Table 3).

### Frequency ratings

The number of categories in “frequency” format questions ranged from three to six. The majority of questions were anchored with either “Not at all”, “None of the time”, “Never” or “No” at one end, and “All of the time” or “Always” at the other. In most questionnaires, the most positive category was the first option presented.

The IVI, with six categories, demonstrated the largest scale range (3.68 logits; Table 3). However, the categories demonstrated uneven distribution of categories (high SD, 0·72). Of the three PROs with five-category response formats, the NEIVFQ had the largest scale range (3.58 logits), but also had uneven category distribution (SD, 0.54). Conversely, the CSS with five categories showed evenly distributed categories (SD 0.38), but a smaller scale range (2.93 logits). The Visual Activity Questionnaire (VAQ) with the five-category format was the best performing PRO instrument in terms of scale range (3.55 logits) and evenness of categories (SD, 0.45). The VAQ has items with simple question format (e.g. item 10; *“I have trouble reading the menu in a dimly lit restaurant”*) and non overlapping categories (*“never, rarely, sometimes, often and always”*). The VSQ with four response categories demonstrated almost comparable coverage of the trait as the five-category format of the VAQ, but demonstrated highly uneven categories (Table 3). Compared to “difficulty” ratings, items rating “frequency” were limited by either a narrow coverage of the trait or unequal width of the categories which might lead to poor differentiation between respondents.

### Severity ratings

Unlike for “difficulty” and “frequency”, there was no uniform response format for “severity” questions. The number of categories varied between three and five. While PROs with four or five categories had a large scale range, the unevenness of the categories was a limiting factor. The CSS with the branching question format (e.g. item 1: “*Are you bothered by double or distorted vision*?” Item 1a: *“If so how bothered are you by double or distorted vision?”*) showed even categories (small SD, 0·08) but demonstrated the smallest scale range (2.89) (Table 4).

### Global ratings

This group represented questions related to global ratings of vision or health. Response categories ranged from three to eight. Questions were formatted with the most positive response option (i.e. “Excellent” and “Perfectly happy”) at one end and the least positive (i.e. “Cannot see at all” and “Poor”) at the other.

The Visual Function and Quality of Life (VF&QOL) questionnaire with a simple question format *(“In general, would you say your vision (with glasses, if you wear them”*) and four response categories (*“very good, good, fair and poor”*) had large scale range (6.20 logits) and very even categories (SD, 0.15). The VSQ with eight categories (VSQ V2 and V3) also had large coverage of the trait with even categories (SD, 0.50) (Table 4). The NEIVFQ (six categories) had the largest scale range (10.18 logits) but the categories were uneven (SD, 0.86). The TyPE questionnaire performed poorly in terms of both scale range (3.89 logits) and evenness of categories (SD, 0.95). Thus, global ratings were best served by four categories, as in the VF&QOL questionnaire (scale range = 6.20 and SD, 0.37). Greater response categories (up to seven) may be used in the formats demonstrated herein.

### The relationship between misfitting items and rating scales

The majority of the items across the PRO instruments with ordered categories fit the Rasch model (Tables 3 and 4). The PRO instruments that demonstrated disordered rating scale categories had higher representation of misfitting items (Table 2). Overall, the PRO instruments with the better fitted items had the better performing rating scale categories in terms of scale measure range and evenness of category utilization (Tables 3 and 4). Among the items demonstrating ordered categories, the Catquest frequency ratings had maximum number of misfitting items (4 out of 7 items) followed by the VF-14 (3 out of 12 items). Notably, the Catquest had very narrow range (2.78) and the VF-14 demonstrated unevenness of category utilization (SD, 1.38) (Table 3). Furthermore, items with the similar content demonstrated acceptable fit statistics with functional rating scales but not with dysfunctional rating scales. For example, the ADVS item 15bc with the content “driving during the day” misfit the Rasch model, conversely, the VDA item 8 and the NEIVFQ item 15c with the similar content perfectly fit the Rasch model. This observation was consistent across other items having similar contents.

The misfitting items from the PRO instruments with dysfunctional rating scales were removed to assess the effect of the item removal on the category functioning. Table 5 shows the threshold values of the items with disordered categories before and after the removal of the misfitting items. Item removal leads to only small changes in threshold values and does not repair disordering of the categories.

**Table 5:** Threshold values of the dysfunctional response categories before and after the removal of the misfitting items

**Questionnaire (Question number)**	**Threshold values (All items)**	**Threshold values (After removing misfitting items)**
IVI (1–19)	−1.45, −1.72, 0.03, 0.72, 2.43	−1.50, −1.73, 0.03, 0.73, 2.48
ADVS (1bc–19bc except 6 and 7bc)	−0.94, −1.00, −0.20, 2.14	−0.60, −1.21, −0.32, 2.13
HVAT (1ab to 10ab)	−0.52, 0.25, −0.04, −1.04, −0.36, 0.26, −0.12, −0.38, 1.96	−0.83, 0.14, −0.17, −0.95, −0.61, 0.33, 0.06, −0.31, 2.35
NEIVFQ (20–25, A12, A13)	−0.07, −0.51, 0.26, 0.33	–0.03, −0.70, 0.11, 0.62

Item removal has little impact on values and does not repair disordered thresholds.

## Discussion

The present study provides a concurrent comparison of the functioning of a wide range of rating scales found in 17 different PROs. Evidence from this study enabled us to formulate an evidence-based guidance for the selection of rating scales for developing new PROs. Although our illustrative examples were drawn from PRO instruments used in ophthalmology, these results may have relevance for other disciplines. However, this should be demonstrated by replication of this work in other disciplines rather than accepting these findings as transferrable.

The results revealed that PROs with a larger number of categories and complicated question formats are more likely to have a dysfunctional rating scale which is also supported by other studies [9,10,41,42]. The Houston Vision Assessment Tool (HVAT) which uses a multiplicative scale (patients make ratings on two scales which are then multiplied to give a more complex final scale) with ten categories demonstrated the highest number of disordered thresholds. The other 10 category scale was also dysfunctional (global ratings of health and vision in the NEIVFQ). However, this may also have been affected by having unlabelled response categories [9,10,43]. The format of questions also plays a vital role in producing dysfunctional rating scale. For example, the ADVS has questions presented in a more complicated, branching style which resulted in poor performance (Table 2). Therefore, fewer, concise, and labelled categories just sufficient to maintain adequate measurement precision (i.e. ability to distinguish between the respondents) would ensure good measurement properties whilst maintaining low respondent burden of the PRO [11,44].

Across the 17 PROs, most of the “difficulty” questions possessed the characteristics (fewer, concise and labelled categories) but not all demonstrated even distribution of categories (Table 3). The VDA demonstrated a superior rating scale performance which is likely due to the design features; an identical four-category format for all questions with conceptually spaces labels, and a simple and uniform question format [45]. While several PROs covered a slightly larger range of the trait, they did so at the sacrifice of equal utilization of categories (i.e. large SD). We found that most of the 5 category scales covered less range than most of the 4 category scales. This illustrates either that more categories can simply add confusion, or that the details of question design and category labelling are also important drivers of rating scale performance than number of categories. The latter conclusion is also supported by the observation of good and bad functioning scales with the same number of response categories (Tables 3 and 4).

Frequency scales did not appear among the dysfunctional sales suggesting people find it easy to respond to frequency ratings. However, “frequency” scales performed less well than ‘difficulty’ scales in terms of both scale range and category evenness (Table 4). An assessment of “severity” scales is difficult given only 4 were included in the study. While two demonstrated excellent range, they suffered from uneven categories. Whereas the one scale with even categories suffered from a limited scale range.

The global rating items were best assessed using a four-category response format, as in the VF&QOL questionnaire, given its high range and even categories. Perhaps the short-description of the categories assisted its good performance. Global ratings with more categories were also functional, the VSQ (seven categories) and the NEIVFQ (five categories) covered a large range and had a fairly even distribution of categories. However, other items in the same instruments, VSQ (eight categories) and NEIVFQ (six categories) had uneven category distribution. Therefore, using more than four or five categories requires careful attention to other attributes of rating scale design. Our findings are also supported by other studies which show that scales with fewer categories out performed the scales with large number of categories [9,46,47].

Items with dysfunctional rating scale categories were more likely to misfit the Rasch model (Table 2). Conversely, the PRO instruments with functional rating scales were likely to have very few misfitting items (Tables 3 and 4). We attempted to remove the misfitting items to determine their effect on disordered categories. We observed that this process alone did not repair disordered categories, however, category widths did expand slightly. Notably, items with similar content fit which used with a functional rating scale but not with a dysfunctional rating scale. This suggests that dysfunctional rating scales add noise to items leading to misfit rather than misfitting items damaging the rating scale. However, the actual interaction between item fit statistics and rating scale category functioning is not clear. This requires further investigation. Given disordered rating scale categories can degrade the psychometric properties of a PRO instrument, a sensible post hoc modification by combining categories is a reasonable remedy. Interested readers are requested to refer to a series of publications by our group which report this approach [23-38].

In this study, we observed that the difficulty ratings provided a wider measurement range and evenness of category utilization than frequency ratings (Table 3). This finding reflects properties of the patient data and suggests that people are better at scaling difficulty than they are at scaling frequency. The reasons for this are unclear but may include frequency of problems rating being confounded by frequency of exposure, which may in turn be confounded by limited access due to cost or other variables. However, this does not mean that difficulty ratings must always be preferred over frequency ratings. We advise PRO instrument developers to exercise their judgement while formulating rating categories on the basis of the construct being measured and the research question (e.g. mental health instruments may require frequency ratings because this is part of the definition of the health state).

Similarly, across each of the attributes under measurement, there are rating scales which perform better in terms of metrics such as measurement range or category evenness. However, the rating scale with the widest range is often not the one with the most even categories i.e. the best rating scale is not clear cut. Therefore, while there is value in these results in informing rating scale selection for new instruments, there remain a number of good choices and judgement must be exercised in selection. A potential limitation of this study was that the population of cataract patients who participated in the study had visual disability in the mild to moderate range of measurement of these instruments. This is because the indications for cataract surgery have shifted towards earlier surgery since most of these PROs were developed [48]. This might have influenced utilization of the more negative end response categories, and thereby may have affected the evenness of categories in certain PROs (e.g. VF-14, VFQ, and VSQ). Despite this issue, many of the rating scales were perfectly functional. Another limitation is that is using existing questionnaires in their native formats means that there are numerous factors that vary across questionnaires–number of categories, category labels, question structure and question wording. These factors were uncontrolled, so were not varied systematically to provide definitive evidence about the influence of these factors on the results. Nevertheless, consistent observations across large numbers of rating scales allows for meaningful conclusions to be drawn.

## Conclusions

Rating scales are fundamental to data collection, and any loss of measurement quality at this level, will degrade the quality of clinical studies. We found that items with simple and uniform question format, four or five and labelled categories are most likely to be functional and often demonstrate characteristics such as hierarchal ordering, even utilization of categories and a good coverage of the latent trait under measurement. On this basis, we have developed guidelines on the design of rating scales. The guidelines may not translate to all situations, but they may represent useful principles for PRO developers.

Evidence-based guidelines for rating scale design

Do’s

Use a maximum of five categories for most ratings (e.g. difficulty, frequency, severity) although up to 7 may work for global ratings.

• Use short descriptors for categories.

• Use non-overlapping categories (e.g. “not at all”, “a little”, “quite a bit” and “a lot”) so that they are mutually exclusive and collectively exhaustive.

• Use a simple question format.

• Use the same response category format for all questions in a domain (as far as possible).

Avoid

• Too many categories.

• Long descriptors for categories.

• Using a neutral category.

• Conceptually over-lapping categories (for e.g. “hardly at all” and “a little”).

• Using a branching question design or other complicated question formats.

• Unlabelled categories (for e.g. 0–10 scale).

## Competing interest

The authors declare that we have no competing interests.

## Authors’ contribution

KP led the research program. KP, VG, and JK undertook the data analysis and interpretation. All authors contributed to writing and revising the manuscript and approved the final manuscript.

## Proprietary interest statement

The authors have no personal financial interest in the development, production, or sale of any device discussed herein.

## Supplementary Material

Additional file 1 Appendix. Seventeen patient-reported outcome questionnaires, their items and response categories used in the study. Click here for file
