# Supplementary material for: The importance of rating scales in measuring patient-reported outcomes
Source: Health Qual Life Outcomes. 2012 Jul 13;10:80. doi: 10.1186/1477-7525-10-80 (PMC3503574; doi:10.1186/1477-7525-10-80)
Supplement: Additional file 1 — Appendix. Seventeen patient-reported outcome questionnaires, their items and response categories used in the study. [file 1477-7525-10-80-S1.doc]

**Appendix 1:** Seventeen patient-reported outcome questionnaires, their items and response categories used in the study.

| **1. Visual Function Index (VFI)** | |
| --- | --- |
| **Item** | **Question format (Response categories)** |
| **VFI 1** | Reading capacities. What are you able to read in the newspaper? (nothing; large printing types; small printing types) |
| **VFI 2** | How good is your distance vision? (good; moderate; poor) |
| **VFI 3** | Are you able to watch television? (yes; no) |
| **VFI 4** | Does your vision prevent you from automobile or bicycle driving? (yes; no) |
| **VFI 5** | Is your vision sufficient for indoor orientation? (sufficient: not sufficient) |
| **VFI 6** | Is your vision sufficient for outdoor orientation? (sufficient: not sufficient) |
| **VFI 7** | Does your vision limit the kind or amount of work or housework you can do? (yes: no) |
| **VFI 8** | Does your vision limit the kind or amount of other activities (e.g. shopping, gardening, hobbies, clubs) you can do? (yes: no) |
| **VFI 9** | Do you need help with eating, dressing, bathing because of your vision? (yes; no) |
| **VFI 10** | Do you need any help from the community to get along in daily life because of your vision? (yes; no) |
| **VFI 11** | Do you need any help from your family to get along in daily life because of your vision? (yes: no) |

| **2. Activities of Daily Living Scale (ADVS)** | |
| --- | --- |
| **Item** | **Question format (Response categories)** |
| **ADVS A** | Have you ever driven a car? (1. Yes (go to 1a); 2. No (go to 3a)) |
| **ADVS 1a** | During the past 3 months, have you driven at night? (1. Yes (go to 1b); 2. No (go to 1c) |
| **ADVS 1b** | Would you say you drive at night with: (5. No difficulty at all (go to 1d); 4. A little difficulty (go to 1d); 3. Moderate difficulty (go to 1d); 2. Extreme difficulty (go to 1d)) |
| **ADVS 1c** | Is it because of your visual problems that you are unable to drive at night? (1. Yes (go to 2a); 2. No (go to 2a)) |
| **ADVS 1d** | How difficult does seeing moving objects such as people or other cars make driving at night for you? (5. No difficulty at all; 4. A little difficult; 3. Moderately difficult; 2. Extremely difficult; 1. So difficult, I no longer drive for this reason) |
| **ADVS 1e** | 1e. How difficult do oncoming headlights or street lights make driving at night for you? (5. No difficulty at all; 4. A little difficult; 3. Moderately difficult; 2. Extremely difficult; 1. So difficult, I no longer drive for this reason) |
| **ADVS 2a** | During the past 3 months, have you been able to drive a car during the day? (1. Yes (go to 2b); 2. No (go to 2c)) |
| **ADVS 2b** | Would you say that you drive during the day with: (PLEASE CHECK ONLY ONE ANSWER) (5. No visual difficulty at all; 4. A little difficulty because of vision; 3. Moderate difficulty because of vision; 2. Extreme difficulty because of vision) |
| **ADVS 2c** | It is because of *visual problems* that you are unable to drive during the day? (1. Yes (go to 3a); 2. No (go to 3a)) |
| **ADVS 2d** | During the past 3 months, have you been able to drive a car in unfamiliar areas? (1. Yes (go to 2e); 2. No (go to 2f) |
| **ADVS 2e** | Would you say that you drive in unfamiliar areas with (PLEASE CHECK ONLY ONE ANSWER) (5. No difficulty at all; 4. A little difficulty; 3. Moderate difficulty; 2. Extreme difficulty) |
| **ADVS 2f** | It is because of *visual problems* that you are unable to drive in unfamiliar areas? (1. Yes (go to 3a); 2. No (go to 3a)) |
| **ADVS 3a** | During the past 3 months, have you tried to read street signs at night either when driving or when you are a passenger in a car? (1. Yes (go to 3b); 2. No (go to 3c) |
| **ADVS 3b** | Would you say that you read street signs at night with: (PLEASE CHECK ONLY ONE ANSWER) (5. No difficulty at all; 4. A little difficulty; 3. Moderate difficulty; 2. Extreme difficulty) |
| **ADVS 3c** | Is it because of *visual problems* that you do not read street signs at night? (1. Yes (go to 4a); 2. No (go to 4a) |
| **ADVS 4a** | During the past 3 months, have you tried to read street signs in daylight? (1. Yes (go to 4b); 2. No (go to 4c)) |
| **ADVS 4b** | Would you say that you read street signs in daylight with: (PLEASE CHECK ONLY ONE ANSWER) (5. No difficulty at all; 4. A little difficulty; 3. Moderate difficulty; 2. Extreme difficulty) |
| **ADVS 4c** | Is it because of *visual problems* that you do not read street signs in daylight? (1. Yes (go to 5a); 2. No (go to 5a) |
| **ADVS 5a** | During the past 3 months, have you used public transportation? (1. Yes (go to 5b); 2. No (go to 5c)) |
| **ADVS 5b** | Would you say that you use public transportation with: (PLEASE CHECK ONLY ONE ANSWER) (5. No visual difficulty at all; 4. A little visual difficulty; 3. Moderate difficulty because of vision; 2. Extreme difficulty because of vision) |
| **ADVS 5c** | Is it because of *visual problems* that you do not use public transportation? (1. Yes (go to 6a); 2. No (go to 6a) |
| **ADVS 6a** | During the past 3 months, have you tried to walk down steps without handrails or help during daylight? (1. Yes (go to 6b); 2. No (go to 6c)) |
| **ADVS 6b** | Would you say that you walk down steps with: (PLEASE CHECK ONLY ONE ANSWER) (5. No apprehension (or fear) at all; 4. A little apprehension (or fear); 3. Moderate apprehension (or fear); 2. Extreme apprehension (or fear)) |
| **ADVS 6c** | Is it because of *visual problems* that you are unable to walk down steps without handrails or help? (1. Yes (go to 7a); 2. No (go to 7a)) |
| **ADVS 7a** | During the past 3 months, have you tried to walk down steps without handrails or help in dim light (or at dusk)? (1. Yes (go to 7b); 2. No (go to 7c)) |
| **ADVS 7b** | Would you say that you walk down steps in dim light with: (PLEASE CHECK ONLY ONE ANSWER) (5. No apprehension (or fear) at all; 4. A little apprehension (or fear); 3. Moderate apprehension (or fear); 2. Extreme apprehension (or fear)) |
| **ADVS 7c** | Is it because of *visual problems* that you are unable to walk down steps in dim light without handrails or help? (1. Yes (go to 8a); 2. No (go to 8a)) |
| **ADVS 8a** | During the past 3 months, on a bright sunny day, can you see people's faces from across the street? (1. Yes (go to 8b); 2. No (go to 8c)) |
| **ADVS 8b** | Would you say that you see faces in bright sunlight with: (PLEASE CHECK ONLY ONE ANSWER) (5. No difficulty at all; 4. A little difficulty; 3. Moderate difficulty; 2. Extreme difficulty) |
| **ADVS 8c** | Is it because of *visual problems* that you are unable to see faces in bright sunlight? (1. Yes (go to 9a); 2. No (go to 9a)) |
| **ADVS 9a** | During the past 3 months, have you watched television? (1. Yes (go to 9b); 2. No (go to 9c)) |
| **ADVS 9b** | Would you say that you are able to see television with: (PLEASE CHECK ONLY ONE ANSWER) (5. No difficulty at all (go to 10a.); 4. A little difficulty (go to 10a); 3. Moderate difficulty (go to 10a); 2. Extreme difficulty (go to 10a)) |
| **ADVS 9c** | Is it because of *visual problems* that you are unable to watch television? (1. Yes (go to 11a); 2. No (go to 11a)) |
| **ADVS 10a** | Can you read number on the television screen? (Yes (go to 10b); (go to 10c)) |
| **ADVS 10b** | Would you say that you are able to read numbers with: (PLEASE CHECK ONLY ONE ANSWER) (No visual difficulty at all; A little visual difficulty because of vision; Moderate visual difficulty because of vision; Extreme difficulty because of vision) |
| **ADVS 10c** | Is it because of *visual problems* that you are unable to read numbers on the television screen? (Yes (go to 11a); No (go to 11a)) |
| **ADVS 11a** | During the past 3 months, have you tried to read the ordinary print in newspapers? ( Yes (go to 11b); No (go to 11c)) |
| **ADVS 11b** | Would you say that you read the ordinary print in newspapers with: (PLEASE CHECK ONLY ONE ANSWER) (No visual difficulty at all; A little visual difficulty because of vision; Moderate visual difficulty because of vision; Extreme difficulty because of vision) |
| **ADVS 11c** | Is it because of *visual problems* that you cannot read the ordinary print in newspapers? (Yes (go to 12a); No (go to 12a)) |
| **ADVS 12a** | During the past 3 months, have you tried to read the directions on medicine bottles? (Yes (go to 12b); No (go to 12c)) |
| **ADVS 12b** | Would you say that you read the directions on medicine bottles with: (PLEASE CHECK ONLY ONE ANSWER) (No visual difficulty at all; A little visual difficulty because of vision; Moderate visual difficulty because of vision; Extreme difficulty because of vision) |
| **ADVS 12c** | Is it because of *visual problems* that you cannot read the directions on medicine bottles? (Yes (go to 13a); No (go to 13a)) |
| **ADVS 13a** | During the past 3 months, have you tried to read the ingredients on cans of food? (Yes (go to 13b); No (go to 13c)) |
| **ADVS 13b** | Would you say that you read the ingredients on cans of food with: (PLEASE CHECK ONLY ONE ANSWER) (No visual difficulty at all; A little visual difficulty because of vision; Moderate visual difficulty because of vision; Extreme difficulty because of vision) |
| **ADVS 13c** | Is it because of *visual problems* that you cannot read the ingredients on cans of food? (Yes (go to 14a); No (go to 14a)) |
| **ADVS 14a** | During the past 3 months, have you been able to write checks without help? (1. Yes (go to 14b); 2. No (go to 14c)) |
| **ADVS 14b** | Would you say that you write checks with: (PLEASE CHECK ONLY ONE ANSWER) (No visual difficulty at all; A little visual difficulty because of vision; Moderate visual difficulty because of vision; Extreme difficulty because of vision) |
| **ADVS 14c** | Is it because of *visual problems* that you cannot write checks without help? (1. Yes (go to 15a); 2. No (go to 15a)) |
| **ADVS 15a** | During the past 3 months, have you tried to thread a needle without using a threading device (or help-mate)? (1. Yes (go to 15b); 2. No (go to 15c)) |
| **ADVS 15b** | Would you say that you thread a needle with: (PLEASE CHECK ONLY ONE ANSWER) (No visual difficulty at all; A little visual difficulty because of vision; Moderate visual difficulty because of vision; Extreme difficulty because of vision) |
| **ADVS 15c** | Is it because of *visual problems* that you have not tried to thread a needle? (1. Yes (go to 16a); 2. No (go to 16a)) |
| **ADVS 16a** | During the past 3 months, have you tried to use rulers, yard sticks or tape measures? (1. Yes (go to 16b); 2. No (go to 16c)) |
| **ADVS 16b** | Would you say that you use rulers, yard sticks or tape measures with: (PLEASE CHECK ONLY ONE ANSWER) (No visual difficulty at all; A little visual difficulty because of vision;. Moderate visual difficulty because of vision; Extreme difficulty because of vision) |
| **ADVS 16c** | Is it because of *visual problems* that you do not use rulers, yard sticks, or tape measures? (1. Yes (go to 17a); 2. No (go to 17a)) |
| **ADVS 17a** | During the past 3 months, have you tried to use a screwdriver? (1. Yes (go to 17b); 2. No (go to 17c)) |
| **ADVS 17b** | Would you say that you use a screwdriver with: (PLEASE CHECK ONLY ONE ANSWER) (No visual difficulty at all; A little visual difficulty because of vision; Moderate visual difficulty because of vision; Extreme difficulty because of vision) |
| **ADVS 17c** | Is it because of *visual problems* that you do not use a screwdriver? (1. Yes (go to 18a); 2. No (go to 18a)) |
| **ADVS 18a** | During the past 3 months, have you prepared meals? (1. Yes (go to 18b); 2. No (go to 18c)) |
| **ADVS 18b** | Would you say that you prepare meals with: (PLEASE CHECK ONLY ONE ANSWER) (No visual difficulty at all; A little visual difficulty because of vision;. Moderate visual difficulty because of vision; Extreme difficulty because of vision) |
| **ADVS 18c** | Is it because of *visual problems* that you do not prepare meals? (1. Yes (go to 19a); 2. No (go to 19a)) |
| **ADVS 19a** | During the past 3 months, have you tried to play cards? (1. Yes (go to 19b); 2. No (go to 19c)) |
| **ADVS 19b** | Would you say that you play cards with: (PLEASE CHECK ONLY ONE ANSWER) (No visual difficulty at all; A little visual difficulty because of vision;. Moderate visual difficulty because of vision; Extreme difficulty because of vision) |
| **ADVS 19c** | Is it because of *visual problems* that you do not play cards? (Yes; No) |

| **3. Visual Activities Questionnaire (VAQ)** | |
| --- | --- |
| **Item** | **Question format (Response categories)** |
| **VAQ 1** | I have a problem adjusting to bright room lighting, after the room lighting has been rather dim. (Never; Rarely; Sometimes; Often; Always) |
| **VAQ 2** | I have trouble noticing things in my peripheral vision. (Never; Rarely; Sometimes; Often; Always) |
| **VAQ 3** | I have trouble finding a specific item on a crowded supermarket shelf. (Never; Rarely; Sometimes; Often; Always) |
| **VAQ 4** | I have problems with lights around me causing glare when I'm trying to see something. (Never; Rarely; Sometimes; Often; Always) |
| **VAQ 5** | I tend to confuse colours (Never; Rarely; Sometimes; Often; Always) |
| **VAQ 6** | I have trouble locating a sign when it is surrounded by a lot of other signs. (Never; Rarely; Sometimes; Often; Always) |
| **VAQ 7** | I have problems reading small print (for example, phone book, newspapers). (Never; Rarely; Sometimes; Often; Always) |
| **VAQ 8** | I have trouble reading a sign or recognising a picture when it's moving, such as an ad on a passing bus or truck. (Never; Rarely; Sometimes; Often; Always) |
| **VAQ 9** | When pouring liquid, I have trouble judging the level of the liquid in a container, such as the level of coffee in a cup. (Never; Rarely; Sometimes; Often; Always) |
| **VAQ 10** | I have trouble reading the menu in a dimly lit restaurant. (Never; Rarely; Sometimes; Often; Always) |
| **VAQ 11** | I have trouble seeing moving objects coming from the side until they are right in front of me. (Never; Rarely; Sometimes; Often; Always) |
| **VAQ 12** | It takes me a long time to adjust to darkness after being in bright light. (Never; Rarely; Sometimes; Often; Always) |
| **VAQ 13** | When I'm driving, other cars surprise me from the side, because I don't notice them until the last moment. (Never; Rarely; Sometimes; Often; Always; Do not drive) |
| **VAQ 14** | I have trouble driving when there are headlights from oncoming cars in my field of view. (Never; Rarely; Sometimes; Often; Always; Do not drive) |
| **VAQ 15** | I have difficulty reading small print under poor lighting. (Never; Rarely; Sometimes; Often; Always) |
| **VAQ 16** | I have problems locating something when it's surrounded by a lot of other things. (Never; Rarely; Sometimes; Often; Always) |
| **VAQ 17** | The colour names that I use disagree with those that other people use. (Never; Rarely; Sometimes; Often; Always) |
| **VAQ 18** | I have problems carrying out activities that require a lot of visual concentration and attention. (Never; Rarely; Sometimes; Often; Always) |
| **VAQ 19** | When I'm walking along, I have trouble noticing objects off to the side. (Never; Rarely; Sometimes; Often; Always) |
| **VAQ 20** | It takes me a long time to find an item in an unfamiliar store. (Never; Rarely; Sometimes; Often; Always) |
| **VAQ 21** | Sometimes when I reach for an object, I find that it is further away (or closer) than I thought. (Never; Rarely; Sometimes; Often; Always; Do not drive) |
| **VAQ 22** | I have difficulty noticing when the car in front of me is speeding up or slowing down. (Never; Rarely; Sometimes; Often; Always) |
| **VAQ 23** | It takes me a long time to adjust to bright sunshine after I have been inside a building for a lengthy period of time. (Never; Rarely; Sometimes; Often; Always) |
| **VAQ 24** | When driving at night, objects from the side unexpectedly appear or pop up in my field of view. (Never; Rarely; Sometimes; Often; Always; Do not drive) |
| **VAQ 25** | I have difficulty distinguishing between colours. (Never; Rarely; Sometimes; Often; Always) |
| **VAQ 26** | I bump into people in a busy store because I have problems seeing them in my peripheral vision. (Never; Rarely; Sometimes; Often; Always) |
| **VAQ 27** | I have difficulty doing any type of work which requires me to see well up close. (Never; Rarely; Sometimes; Often; Always) |
| **VAQ 28** | I have trouble adjusting from bright to dim lighting, such as when going from daylight into a dark movie theatre. (Never; Rarely; Sometimes; Often; Always) |
| **VAQ 29** | When driving at night in the rain, I have difficulty seeing the road because of headlights from oncoming cars. (Never; Rarely; Sometimes; Often; Always; Do not drive) |
| **VAQ 30** | When riding in a car, other cars on the road seem to be going too fast. (Never; Rarely; Sometimes; Often; Always) |
| **VAQ 31** | I find it difficult changing lanes in traffic because I have trouble seeing cars in the next lane. (Never; Rarely; Sometimes; Often; Always; Do not drive) |
| **VAQ 32** | I have problems judging how close or far things are from me. (Never; Rarely; Sometimes; Often; Always) |
| **VAQ 33** | It takes me a long time to get acquainted with new surroundings. (Never; Rarely; Sometimes; Often; Always) |

| **4. Cataract Symptom Score (CSS)** | |
| --- | --- |
| **Item** | **Question format (Response categories)** |
| **CSS 1** | Are you bothered by double or distorted vision? (Yes: No) |
| **CSS 1a** | If so how bothered are you by double or distorted vision? (Very bothered: Somewhat bothered; A little bothered) |
| **CSS 2** | Are you bothered by seeing glare, halo or rings around light? (Yes; No) |
| **CSS 2a** | If so how bothered are you by seeing glare, halo or rings around light? (Very bothered: Somewhat bothered; A little bothered) |
| **CSS 3** | Are you bothered by blurring vision? (Yes; No) |
| **CSS 3a** | If so how bothered are you by blurry vision? (Very bothered: Somewhat bothered; A little bothered) |
| **CSS 4** | Are you bothered by colours looking different to how they used to in a way that is disturbing? (Yes; No) |
| **CSS 4a** | If so how bothered are you by colours looking different to how they used to in a way that is disturbing? (Very bothered: Somewhat bothered; A little bothered) |
| **CSS 5** | Are you bothered by worsening of vision in the last month? (Yes; No) |
| **CSS 5a** | If so how bothered are you by worsening of vision in the last month? (Very bothered: Somewhat bothered; A little bothered) |

| **5. Visual Function (VF-14)** | | | |
| --- | --- | --- | --- |
| **Item** | | **Question format (Response categories)** | |
| **VF14_1** | | Do you have difficulty, even with glasses, reading small print, such as labels on medicine bottles, a telephone book, food labels? (Yes/No/Not applicable) (A little; A moderate amount; A great deal, Are you unable to do the activity) | |
| **VF14_2** | | Do you have difficulty, even with glasses, reading a newspaper or a book? (Yes/No/Not applicable) If yes, how much difficulty do you currently have? (A little; A moderate amount; A great deal, Are you unable to do the activity) | |
| **VF14_3** | | Do you have difficulty, even with glasses, reading a large-print book or large-print newspaper or numbers on a telephone? (Yes/No/Not applicable) If yes, how much difficulty do you currently have? (A little; A moderate amount; A great deal, Are you unable to do the activity) | |
| **VF14_4** | | Do you have difficulty, even with glasses, recognising people when they are close to you? (Yes/No/Not applicable) If yes, how much difficulty do you currently have? (A little; A moderate amount; A great deal, Are you unable to do the activity) | |
| **VF14_5** | | Do you have difficulty, even with glasses, seeing steps, stairs, or curbs? (Yes/No/Not applicable) If yes, how much difficulty do you currently have? (A little; A moderate amount; A great deal, Are you unable to do the activity) | |
| **VF14_6** | | Do you have difficulty, even with glasses, reading traffic signs, street signs, or store signs? (Yes/No/Not applicable) If yes, how much difficulty do you currently have? (A little; A moderate amount; A great deal, Are you unable to do the activity) | |
| **VF14_7** | | Do you have difficulty, even with glasses, doing fine handwork like sewing, knitting, crocheting, and carpentry? (Yes/No/Not applicable) If yes, how much difficulty do you currently have? (A little; A moderate amount; A great deal, Are you unable to do the activity) | |
| **VF14_8** | | Do you have difficulty, even with glasses, writing cheques or filling out forms? (Yes/No/Not applicable) If yes, how much difficulty do you currently have? (A little; A moderate amount; A great deal, Are you unable to do the activity) | |
| **VF14_9** | | Do you have difficulty, even with glasses, playing games such as bingo, dominos, card games, mahjong? (Yes/No/Not applicable) If yes, how much difficulty do you currently have? (A little; A moderate amount; A great deal, Are you unable to do the activity) | |
| **VF14_10** | | Do you have difficulty, even with glasses, taking part in sports like bowling, handball, tennis, golf? (Yes/No/Not applicable) If yes, how much difficulty do you currently have? (A little; A moderate amount; A great deal, Are you unable to do the activity) | |
| **VF14_11** | | Do you have difficulty, even with glasses, cooking? (Yes/No/Not applicable) If yes, how much difficulty do you currently have? (A little; A moderate amount; A great deal, Are you unable to do the activity) | |
| **VF14_12** | | Do you have difficulty, even with glasses, watching television? (Yes/No/Not applicable) If yes, how much difficulty do you currently have? (A little; A moderate amount; A great deal, Are you unable to do the activity) | |
|  | | Do you currently drive a car? (Yes (go to Q14); No (go to Q16)) | |
| **VF14_13** | | How much difficulty do you have driving during the day because of your vision? Do you have: : (No difficulty; A little difficulty; A moderate amount of difficulty; A great deal of difficulty) | |
| **VF14_14** | | How much difficulty do you have driving at night because of your vision? Do you have: (No difficulty; A little difficulty; A moderate amount of difficulty; A great deal of difficulty) | |
| **VF14_15** | | Have you ever driven a car? (Yes (go to Q17); No (stop)) | |
| **VF14_16** | | When did you stop driving? (Less than 6 months ago; 6-12 months ago; More than 12 months ago) | |
| **VF14_17** | | Why did you stop driving? (Vision; Other illness; Other reason) | |
| **6. CatQuest (CATQ)** | | |  |
| **Item** | **Question format (Response categories)** | |  |
| **CATQ 1** | Do you normally read a newspaper? (No: Yes) | |  |
| **CATQ 1a** | If yes, do you normally read… (one newspaper a week at the most; one newspaper a day(approx); several newspapers a day) | |  |
| **CATQ 1b** | If you normally do not read a newspaper, is it only because of poor vision? (Yes; No) | |  |
| **CATQ 2** | Do you normally buy your nondurable goods yourself or regularly make other purchases? (No; Yes) | |  |
| **CATQ 2a** | If yes, do you normally shop…(once a week at the most; 2-4 times a week (approx); Daily) | |  |
| **CATQ 2b** | If you normally do not shop yourself, is it only because of poor vision? (Yes; No) | |  |
| **CATQ 3** | Do you normally talk a walk outside on your own or with company? (No, never; Yes) | |  |
| **CATQ 3a** | If yes, how often… (once a week at the most; 2-4 times a week (approx); Daily) | |  |
| **CATQ 3b** | If you never take a walk outside, is it only because of poor vision? (Yes; No) | |  |
| **CATQ 4** | Do you normally do needlework, woodwork, embroidery or other handicrafts? (No, never; Yes) | |  |
| **CATQ 4a** | If yes, how often… (once a week at the most; 2-4 times a week (approx); Daily) | |  |
| **CATQ 4b** | If you normally do not do needlework or other handicrafts, is it only because of poor vision? (Yes; No) | |  |
| **CATQ 5** | Do you normally watch television? (No, never; Yes) | |  |
| **CATQ 5a** | If yes, how often… (once a week at the most; one hour daily (approx); several hours daily) | |  |
| **CATQ 5b** | If you normally do not watch television, is it only because of poor vision? (Yes; No) | |  |
| **CATQ 6** | Do you have another hobby or leisure activity that you would like to do? (No; Yes) Hobby/activity……………….. | |  |
| **CATQ 6a** | If you have answered yes to this question, how often do you perform this activity? (Once a week at the most; 2-4 times a week (approx); Daily) | |  |
|  | **Because of your vision, do you have difficulty with the following activities? If yes how much?** | |  |
| **CATQ 7** | Read newspaper print… (Yes, extreme difficulty; Yes, much difficulty; Yes, some difficulty; No, no difficulty; Cannot say) | |  |
| **CATQ 8** | Recognise the faces of people you meet… (Yes, extreme difficulty; Yes, much difficulty; Yes, some difficulty; No, no difficulty; Cannot say) | |  |
| **CATQ 9** | See the prices of goods when you shop...(Yes, extreme difficulty; Yes, much difficulty; Yes, some difficulty; No, no difficulty; Cannot say) | |  |
| **CATQ 10** | See to walk on uneven ground...(Yes, extreme difficulty; Yes, much difficulty; Yes, some difficulty; No, no difficulty; Cannot say) | |  |
| **CATQ 11** | See to do needlework… (Yes, extreme difficulty; Yes, much difficulty; Yes, some difficulty; No, no difficulty; Cannot say) | |  |
| **CATQ 12** | Read television text...(Yes, extreme difficulty; Yes, much difficulty; Yes, some difficulty; No, no difficulty; Cannot say) | |  |
| **CATQ 13** | See to carry out the activity / hobby you named previously..(Yes, extreme difficulty; Yes, much difficulty; Yes, some difficulty; No, no difficulty; Cannot say) | |  |
| **CATQ 14** | Does your present vision in any way give you problems in your daily life? (Yes, extreme difficulty; Yes, much difficulty; Yes, some difficulty; No, no difficulty; Cannot say) | |  |
| **CATQ 15** | Are you satisfied or dissatisfied with your present vision? (Very dissatisfied; rather dissatisfied; rather satisfied; very satisfied; cannot say) | |  |
| **CATQ 16** | Different lighting conditions (darkness, rain) can sometimes influence one's vision, producing visual disturbances such as glare or dazzling. Do you think that headlights, lamps, sunlight, and other lights reduce your vision more often now than before? ( | |  |
| **CATQ 16a** | If yes, does it give you…. (Extreme difficulty; much difficulty; some difficulty; no difficulty; Cannot say) | |  |
| **CATQ 17** | In persons with cataract, great visual differences between the two eyes can occur. This can lead to poor depth perception so that you may, for example, spill when pouring liquids. If one eye is already operated on, one can experience great differences in clarity and colour between two eyes. Do you experience visual disturbances from any of the above named difference between two eyes? (Yes; No) | |  |
| **CATQ 17a** | If yes, does it give you…. (Extreme difficulty; much difficulty; some difficulty; no difficulty; Cannot say) | |  |
| **CATQ 18** | Do you have any illness for which you take medicine regularly? (No; Yes, one illness; Yes, more than one illness; Cannot say) | |  |
| **CATQ 19** | Do you have help in your home (other than from those living in your home)? (Yes, help from a friend/ relative; Yes, home help; Yes, from staff at the aged persons home/ nursing home / hospital; cannot say) | |  |
| **CATQ 19a** | If you have daily help, how may hours per day?............hours/day | |  |
| **CATQ 19b** | If you have help each week, how many hours per week ………hours/week | |  |
| **CATQ 20** | Do you have subsidized travel by taxi? (No; Yes) | |  |
| **CATQ 21** | (Do you have employment? No; Yes) | |  |
| **CATQ 21a** | If yes, are you off sick? (No; Yes) | |  |
| **CATQ 22** | Do you live alone (No; Yes) | |  |
| **CATQ 23** | Have you driven a car during the past 12 months? (No; Yes) | |  |
| **CATQ 23a** | If yes, what is the situation at present….(Drive both during day and at night; Drive only in daylight; Have given up driving because of poor vision; Have given up driving for other reasons) | |  |
| **CATQ 24** | If you still drive a car, does your vision give you difficulty while you are driving? (Yes, extreme difficulty; Yes, much difficulty; Yes, some difficulty; No, no difficulty; Cannot say) | |  |

| **7. Visual Function and Quality of Life (VFQOL)** | |
| --- | --- |
| **Item** | **Question format (Response categories)** |
| **VFQOL 1** | In general, would you say your vision (with glasses, if you wear them) is: (Very good; Good; Fair; Poor) |
| **VFQOL 2** | To what extent does your sight limit you in your daily activities? (Not at all; A little; Quite a lot; A lot |
| **VFQOL 3** | How much problem do you have recognising people across the street? (Not at all; A little; Quite a lot; A lot |
| **VFQOL 4** | How much problem do you have recognising the face of a person standing near you? (Not at all; A little; Quite a lot; A lot |
| **VFQOL 5** | How much problem do you have recognising small or minute objects (such as grains or the lines in your hand)? (Not at all; A little; Quite a lot; A lot |
| **VFQOL 6** | When you are walking along, how much problem do you have noticing objects off to the side? (Not at all; A little; Quite a lot; A lot |
| **VFQOL 7a** | How much problem do you have adjusting to darkness after being in a bright light? (Not at all; A little; Quite a lot; A lot |
| **VFQOL 7b** | How much problem do you have adjusting to brightness after being in a dark place? (Not at all; A little; Quite a lot; A lot |
| **VFQOL 8** | How much problem do you have locating something when it is surrounded by a lot of other things (like finding a specific food item on your plate)? (Not at all; A little; Quite a lot; A lot) |
| **VFQOL 9** | How much problem do you have in recognising colours? (Not at all; A little; Quite a lot; A lot) |
| **VFQOL 10** | When you reach for an object (e.g. to take a glass), how much problem do you have in finding it, because it is further away or closer than you thought? (Not at all; A little; Quite a lot; A lot) |
| **VFQOL 11a** | How much problem do you have in recognising a person when you are in bright light? (Not at all; A little; Quite a lot; A lot) |
| **VFQOL 11b** | How much problem do you have seeing with bright lights shining on your eyes (such as from an oncoming bus or car)? (Not at all; A little; Quite a lot; A lot) |
| **VFQOL 12** | How much problem do you have because of your vision in doing the following activities unaided…… |
| **VFQOL 12a** | Bathing? (Not at all; A little; Quite a lot; A lot) |
|  | Does someone help you? Yes; No |
| **VFQOL 12b** | Eating? (Not at all; A little; Quite a lot; A lot) |
|  | Does someone help you? (Yes; No) |
| **VFQOL 12c** | Dressing? (Not at all; A little; Quite a lot; A lot) |
|  | Does someone help you? (Yes; No) |
| **VFQOL 12d** | Toileting? (Not at all; A little; Quite a lot; A lot) |
|  | Does someone help you? (Yes; No) |
| **VFQOL 13** | How much problem do you have because of your vision in doing the following activities unaided…… |
| **VFQOL 13a** | Walking to neighbours? (Not at all; A little; Quite a lot; A lot) |
|  | Does someone help you? (Yes; No) |
| **VFQOL 13b** | Walking to shops? (Not at all; A little; Quite a lot; A lot) |
|  | Does someone help you? (Yes; No) |
| **VFQOL 13c** | Doing your usual household chores? (Not at all; A little; Quite a lot; A lot) |
|  | Does someone help you? (Yes; No) |
| **VFQOL 14** | Because of your visual problems, do you feel less inclined to participate in the following…... |
| **VFQOL 14a** | Attending social functions like weddings, funerals, festivals? (Not at all; A little; Quite a lot; A lot) |
|  | Does someone help you? (Yes; No) |
| **VFQOL 14b** | Meeting with friends and relatives? (Not at all; A little; Quite a lot; A lot) |
|  | Does someone help you? (Yes; No) |
| **VFQOL 15** | Because of your vision problems do you feel…. |
| **VFQOL 15a** | A burden on others? (Not at all; A little; Quite a lot; A lot) |
| **VFQOL 15b** | Dejected? (Not at all; A little; Quite a lot; A lot) |
| **VFQOL 15c** | Loss of confidence in doing usual activities? (Not at all; A little; Quite a lot; A lot) |

| **8. Quality of Life Visual Function (QOLVF)** | |
| --- | --- |
| **Item** | **Question format/ Response categories** |
| **QOLVF 1** | Does the quality of your vision prevent you from performing your ordinary daily activities normally? (Not at all; Quite a lot; Very much) |
| **QOLVF 2** | Are you unhappy about your visual condition? (Not at all; Quite a lot; Very much) |
| **QOLVF 3** | How much are you concerned with a possible worsening of your visual condition? (Not at all; Quite a lot; Very much) |
| **QOLVF 4** | Because of your visual problems do you feel less inclined to meet people, friends/ relatives? (Not at all; Quite a lot; Very much) |
| **QOLVF 5** | Because of your visual problems do you feel useless or a burden to others? (Not at all; Quite a lot; Very much) |
| **QOLVF 6** | Because of your vision do you have problems crossing the street? (Not at all; Quite a lot; Very much) |
| **QOLVF 7** | Because of your vision do you bump against other people when in crowded areas? (Not at all; Quite a lot; Very much) |
| **QOLVF 8** | Because of your vision do you have problems in perceiving a dip on the ground or a step? (Not at all; Quite a lot; Very much) |
| **QOLVF 9** | Because of your vision do you have problems in reading prices in a shop window? (Not at all; Quite a lot; Very much) |
| **QOLVF 10** | Because of your vision do you have problems in recognizing people across the street? (Not at all; Quite a lot; Very much) |
| **QOLVF 11** | Because of your vision do you have problems in recognizing a person in a crowded room? (Not at all; Quite a lot; Very much) |
| **QOLVF 12** | Because of your vision do you have problems in reading an article in a newspaper or names/ numbers in the telephone directory? (Not at all; Quite a lot; Very much) |
| **QOLVF 13** | Because of your vision do you have problems in doing a manual activity such as cooking, sewing, cutting your nails? (Not at all; Quite a lot; Very much) |
| **QOLVF 14** | Does your vision deteriorate in bright light (for instance on a sunny day)? (Not at all; Quite a lot; Very much) |
| **QOLVF 15** | Does your vision deteriorate in dim light (for instance at dusk)? (Not at all; Quite a lot; Very much) |
| **QOLVF 16** | Do you have a driving licence? (Yes; No) |
| **QOLVF 16a** | If "YES": how much is your driving disturbed by the lights of oncoming cars? (Not at all; Quite a lot; Very much) |
| **QOLVF 16b** | If "NO": how much is your vision disturbed by the lights of oncoming cars? (Not at all; Quite a lot; Very much) |
| **QOLVF 17** | How much problem do you have in recognising colours? (Not at all; Quite a lot; Very much) |

| **9. Visual Disability Assessment (VDA)** | |
| --- | --- |
| **Item** | **Question format (Response categories)** |
| **VDA 1** | To what extent, if at all, does your vision interfere with your ability to read? (Not at all; A little; Quite a bit; A lot; Not applicable = 'no score') |
| **VDA 2** | To what extent, if at all, does your vision interfere with your ability to see in the distance? (Not at all; A little; Quite a bit; A lot; Not applicable = 'no score') |
| **VDA 3** | To what extent, if at all, does your vision interfere with your ability to recognise faces across the street? (Not at all; A little; Quite a bit; A lot; Not applicable = 'no score') |
| **VDA 4** | To what extent, if at all, does your vision interfere with your ability to watch TV? (Not at all; A little; Quite a bit; A lot; Not applicable = 'no score') |
| **VDA 5** | To what extent, if at all, does your vision interfere with your ability to see in bright light/ glare? (Not at all; A little; Quite a bit; A lot; Not applicable = 'no score') |
| **VDA 6** | To what extent, if at all, does your vision interfere with your ability to see in poor or dim light? (Not at all; A little; Quite a bit; A lot; Not applicable = 'no score') |
| **VDA 7** | To what extent, if at all, does your vision interfere with your ability to appreciate colours? (Not at all; A little; Quite a bit; A lot; Not applicable = 'no score') |
| **VDA 8** | To what extent, if at all, does your vision interfere with your ability to drive a car by day? (Not at all; A little; Quite a bit; A lot; Not applicable = 'no score') |
| **VDA 9** | To what extent, if at all, does your vision interfere with your ability to drive a car by night? (Not at all; A little; Quite a bit; A lot; Not applicable = 'no score') |
| **VDA 10** | To what extent, if at all, does your vision interfere with your ability to walk around inside? (Not at all; A little; Quite a bit; A lot; Not applicable = 'no score') |
| **VDA 11** | To what extent, if at all, does your vision interfere with your ability to walk around outside? (Not at all; A little; Quite a bit; A lot; Not applicable = 'no score') |
| **VDA 12** | To what extent, if at all, does your vision interfere with your ability to use steps? (Not at all; A little; Quite a bit; A lot; Not applicable = 'no score') |
| **VDA 13** | To what extent, if at all, does your vision interfere with your ability to cross the road? (Not at all; A little; Quite a bit; A lot; Not applicable = 'no score') |
| **VDA 14** | To what extent, if at all, does your vision interfere with your ability to use public transport? not at all; a little; quite a bit; a lot; not applicable = 'no score') |
| **VDA 15** | To what extent, if at all, does your vision interfere with your ability to travel independently? (Not at all; A little; Quite a bit; A lot; Not applicable = 'no score') |
| **VDA 16** | To what extent, if at all, does your vision interfere with your ability to move in unfamiliar surroundings? (Not at all; A little; Quite a bit; A lot; Not applicable = 'no score') |
| **VDA 17** | To what extent, if at all, does your vision interfere with your ability to do your employment/ housework activities? (Not at all; A little; Quite a bit; A lot; Not applicable = 'no score') |
| **VDA 18** | To what extent, if at all, does your vision interfere with your ability to do your hobbies/leisure activities (Not at all; A little; Quite a bit; A lot; Not applicable = 'no score') |

| **10. Vision Core Measure 1 (VCM 1)** | |
| --- | --- |
| **Item** | **Question format (Response categories)** |
| **VCM1_1** | In the past month, How often has your eyesight made you concerned or worried about your general safety at home? (Not at all; Very rarely; A little of the time; A fair amount of the time; A lot of the time; All the time) |
| **VCM1_2** | In the past month, How often has your eyesight made you concerned or worried about your general safety outside of your home? (Not at all; Very rarely; A little of the time; A fair amount of the time; A lot of the time; All the time) |
| **VCM1_3** | In the past month, How often has your eyesight stopped you from doing the things you want to do? (Not at all; Very rarely; A little of the time; A fair amount of the time; A lot of the time; All the time) |
| **VCM1_4** | In the past month, Have you felt embarrassed because of your eyesight? (Not at all; Very rarely; A little of the time; A fair amount of the time; A lot of the time; All the time) |
| **VCM1_5** | In the past month, Have you felt frustrated or annoyed because of your eyesight? (Not at all; Very rarely; A little of the time; A fair amount of the time; A lot of the time; All the time) |
| **VCM1_6** | In the past month, Have you felt lonely or isolated because of your eyesight? (Not at all; Very rarely; A little of the time; A fair amount of the time; A lot of the time; All the time) |
| **VCM1_7** | In the past month, Have you felt sad or low because of your eyesight? (Not at all; Very rarely; A little of the time; A fair amount of the time; A lot of the time; All the time) |
| **VCM1_8** | In the past month, How often have you worried about your eyesight getting worse? (Not at all; Very rarely; A little of the time; A fair amount of the time; A lot of the time; All the time) |
| **VCM1_9** | In the past month, How often has your eyesight made you concerned or worried about coping with everyday life? (Not at all; Very rarely; A little of the time; A fair amount of the time; A lot of the time; All the time) |
| **VCM1_10** | In the past month, How much has your eyesight interfered with your life in general? (Not at all; Very rarely; A little of the time; A fair amount of the time; A lot of the time; All the time) |

| **11. Cataract Symptom Score (CSS)** | |
| --- | --- |
| **Item** | **Question format (Response categories)** |
| **CSS 1** | Are you bothered by double or distorted vision? (Yes; No) |
| **CSS 1a** | If so how bothered are you by double or distorted vision? (Very bothered; Somewhat bothered; A little bothered) |
| **CSS 2** | Are you bothered by seeing glare, halo or rings around light? (Yes; No) |
| **CSS 2a** | If so how bothered are you by seeing glare, halo or rings around light? (Very bothered; Somewhat bothered; A little bothered) |
| **CSS 3** | Are you bothered by blurring vision? (Yes; No) |
| **CSS 3a** | If so how bothered are you by blurry vision? (Very bothered; Somewhat bothered; A little bothered) |
| **CSS 4** | Are you bothered by colours looking different to how they used to in a way that is disturbing? (Yes; No) |
| **CSS 4a** | If so how bothered are you by colours looking different to how they used to in a way that is disturbing? (Very bothered; Somewhat bothered; A little bothered) |
| **CSS 5** | Are you bothered by worsening of vision in the last month? (Yes; No) |
| **CSS 5a** | If so how bothered are you by worsening of vision in the last month? (Very bothered; Somewhat bothered; A little bothered) |

| **12. Impact of Cataract Surgery (ICS)** | |
| --- | --- |
| **Item** | **Question format (Response categories)** |
| **ICS 1** | Can you read ordinary newspaper-size print? (Yes; No) |
| **ICS 1a** | If YES, what visual aids do you need to be able to read? (none; spectacles; hand-held or stand magnifiers; others (please specify)) |
| **ICS 2** | Do you experience any visual problems while watching TV due to your cataractous eye? (Yes, difficulties; No) |
| **ICS 3** | Do you experience difficulties when orientating in unfamiliar surroundings? (No problems; Some problems; Severe problems) |
| **ICS 4a** | Do you experience difficulties in estimating distance nearby (e.g. pouring a cup of coffee)? (Yes, difficulties; No) |
| **ICS 4b** | Do you experience difficulties in estimating distance far away (e.g. while driving (drivers) or in other ‘traffic situations’)? (Yes, difficulties; No) |

| **13. Technology of Patient Experience (TyPE)** | |
| --- | --- |
| **Item** | **Question format (Response categories)** |
| **T 1** | How would you rate you vision? (How well do you see?) (Poor; Fair; Good; Very good; Excellent) |
|  | **How much does your vision hinder, limit, or disable you in each of the following activities?** |
| **T 2** | Your usual activities.. (Not at all; A little bit; Some; Quite a lot; Totally disabled; Don’t do for other reasons= no score) |
| **T 3** | Recognising people or objects across the street... (Not at all; A little bit; Some; Quite a lot; Totally disabled; Don’t do for other reasons= no score) |
| **T 4** | Reading price labels in shops and supermarkets.. (Not at all; A little bit; Some; Quite a lot; Totally disabled; Don’t do for other reasons= no score) |
| **T 5** | Reading a magazine, newspaper or book... (Not at all; A little bit; Some; Quite a lot; Totally disabled; Don’t do for other reasons= no score) |
| **T 6** | Knitting or sewing…. (Not at all; A little bit; Some; Quite a lot; Totally disabled; Don’t do for other reasons= no score) |
| **T 7** | Watching television…(not at all = 1; a little bit = 2; some = 3; quite a lot = 4; totally disabled = 5; don’t do for other reasons = 0) |
| **T 8** | Daytime driving... (Not at all; A little bit; Some; Quite a lot; Totally disabled; Don’t do for other reasons= no score) |
| **T 9** | Night-time driving... (Not at all; A little bit; Some; Quite a lot; Totally disabled; Don’t do for other reasons= no score) |
|  | How much are you hindered, limited or disabled by glare (dazzling light) in each of the following activities? |
| **T 10** | Your usual activities. (Not at all; A little bit; Some; Quite a lot; Totally disabled; Don’t do for other reasons= no score) |
| **T 11** | Reading shiny paper (such as a magazine) (Not at all; A little bit; Some; Quite a lot; Totally disabled; Don’t do for other reasons= no score) |
| **T 12** | Driving towards the sun or oncoming headlights (Not at all; A little bit; Some; Quite a lot; Totally disabled; Don’t do for other reasons= no score) |
| **T 13** | Walking outside on a sunny day (Not at all; A little bit; Some; Quite a lot; Totally disabled; Don’t do for other reasons= no score) |
| **T 14** | Who filled in this form? (I filled it out with no help; I filled it out with help from family and friends; I filled it out with help form a nurse or doctor or Bucks Association for Blind (BAB) volunteer; Family and friends filled it out) |
| **T 15** | Have you had a recent illness, injury, or emotional upset that has affected how you answer these questions? (Yes; No) |

| **14. The Houston Vision Assessment Test (HVAT)** | |
| --- | --- |
| **Item** | **Question format (Response categories)** |
| **HVAT 1a** | To what extent is your cooking impaired? (Not at all limited; Slightly limited; Somewhat limited; Moderately limited; Severely limited; I do not cook = 'no score') |
| **HVAT 1b** | If there are limitations, how much is because of eyesight? (I have no visual or other physical limitations; None due to eyesight; Some due to eyesight; Half due to eyesight; Most due to eyesight; All due to eyesight) |
| **HVAT 2a** | To what extent is your driving at night impaired by oncoming headlights? (Not at all limited; Slightly limited; Somewhat limited; Moderately limited; Severely limited; I have never driven = 'no score') |
| **HVAT 2b** | If there are limitations, how much is because of eyesight? (I have no visual or other physical limitations; None due to eyesight; Some due to eyesight; Half due to eyesight; Most due to eyesight; All due to eyesight) |
| **HVAT 3a** | To what extent is your driving during the day impaired? (Not at all limited; Slightly limited; somewhat limited; Moderately limited; Severely limited; I have never driven = 'no score') |
| **HVAT 3b** | If there are limitations, how much is because of eyesight? (I have no visual or other physical limitations; None due to eyesight; Some due to eyesight; Half due to eyesight; Most due to eyesight; All due to eyesight) |
| **HVAT 4a** | To what extent is your housework impaired? (Not at all limited; Slightly limited; Somewhat limited; Moderately limited; Severely limited; I do not do housework = 'no score') |
| **HVAT 4b** | If there are limitations, how much is because of eyesight? (I have no visual or other physical limitations; None due to eyesight; Some due to eyesight; Half due to eyesight; Most due to eyesight; All due to eyesight) |
| **HVAT 5a** | To what extent are your leisure activities impaired? (Not at all limited; slightly limited; somewhat limited; Moderately limited; Severely limited; I am not involved in leisure activities = 'no score') |
| **HVAT 5b** | If there are limitations, how much of this is because of eyesight? (I have no visual or other physical limitations; None due to eyesight; Some due to eyesight; Half due to eyesight; Most due to eyesight; All due to eyesight) |
| **HVAT 6a** | To what extent are your outdoor activities impaired? (not at all limited; slightly limited; somewhat limited; Moderately limited; Severely limited; I am not involved in outdoor activities = 'no score') |
| **HVAT 6b** | If there are limitations, how much is because of eyesight? (I have no visual or other physical limitations; none due to eyesight; some due to eyesight; half due to eyesight ; most due to eyesight; all due to eyesight) |
| **HVAT 7a** | To what extent is your reading impaired? (Not at all limited; Slightly limited; Somewhat limited; moderately limited; Severely limited; I do not read = 'no score') |
| **HVAT 7b** | If there are limitations, how much is because of eyesight? (I have no visual or other physical limitations; None due to eyesight; Some due to eyesight; Half due to eyesight ; Most due to eyesight; All due to eyesight) |
| **HVAT 8a** | To what extent is your taking medication impaired? (not at all limited; slightly limited; somewhat limited; moderately limited; severely limited; I do not take medication = 'no score') |
| **HVAT 8b** | If there are limitations, how much is because of eyesight? (I have no visual or other physical limitations; None due to eyesight; Some due to eyesight; Half due to eyesight; Most due to eyesight; All due to eyesight) |
| **HVAT 9a** | To what extent is your watching TV impaired? (not at all limited; slightly limited; somewhat limited; moderately limited; severely limited; I do not take medication = 'no score') |
| **HVAT 9b** | If there are limitations, how much is because of eyesight? (I have no visual or other physical limitations; None due to eyesight; Some due to eyesight; Half due to eyesight; Most due to eyesight; All due to eyesight) |
| **HVAT 10a** | To what extent is your writing impaired? (Not at all limited; slightly limited; Somewhat limited; moderately limited; Severely limited; I do not take medication = 'no score') |
| **HVAT 10b** | If there are limitations, how much is because of eyesight? (I have no visual or other physical limitations; None due to eyesight; Some due to eyesight; Half due to eyesight; Most due to eyesight; All due to eyesight) |
| **HVAT 11** | How certain do you feel about all the answers you gave? (Not at all certain; certain on some of the answers; Certain on about half the answers; Certain on most of the answers; I feel very certain on all of the answers) |

| **15. Impact of Visual Impairment (IVI)** | |
| --- | --- |
| **Item** | **Question format (Response categories)** |
| **IVI 1** | In the past month, how much has your eyesight interfered with paid or voluntary work? (Not at all; Hardly at all; A little; A fair amount; A lot; Can't do because of eyesight; Don't do this for other reasons= no score) |
| **IVI 2** | In the past month, how much has your eyesight interfered with favourite pastimes or hobbies? (Not at all; Hardly at all; A little; A fair amount; A lot; Can't do because of eyesight; Don't do this for other reasons= no score) |
| **IVI 3** | In the past month, how much has your eyesight interfered with your ability to see and enjoy TV? Not at all; Hardly at all; A little; A fair amount; A lot; Can't do because of eyesight; Don't do this for other reasons= no score) |
| **IVI 4** | In the past month, how much has your eyesight interfered with taking part in recreational activities such as bowling, walking or golf? (Not at all; Hardly at all; A little; A fair amount; A lot; Can't do because of eyesight; Don't do this for other reasons= no score) |
| **IVI 5** | In the past month, how much has your eyesight interfered with going out to sports events, movies or plays? (Not at all; Hardly at all; A little; A fair amount; A lot; Can't do because of eyesight; Don't do this for other reasons= no score) |
| **IVI 6** | In the past month, how much has your eyesight interfered with shopping? (finding what you want and paying for it) (Not at all; Hardly at all; A little; A fair amount; A lot; Can't do because of eyesight; Don't do this for other reasons= no score) |
| **IVI 7** | In the past month, how much has your eyesight interfered with reading ordinary size print? (for example newspapers) (Not at all; Hardly at all; A little; A fair amount; A lot; Can't do because of eyesight; Don't do this for other reasons= no score) |
| **IVI 8** | In the past month, how much has your eyesight interfered with visiting friends or family? (Not at all; Hardly at all; A little; A fair amount; A lot; Can't do because of eyesight; Don't do this for other reasons= no score) |
| **IVI 9** | In the past month, how much has your eyesight interfered with recognising or meeting people? (Not at all; Hardly at all; A little; A fair amount; A lot; Can't do because of eyesight; Don't do this for other reasons= no score) |
| **IVI 10** | In the past month, how much has your eyesight interfered with getting information that you need? (Not at all; Hardly at all; A little; A fair amount; A lot; Can't do because of eyesight; Don't do this for other reasons= no score) |
| **IVI 11** | In the past month, how much has your eyesight interfered with generally looking after your appearance? (face, hair, clothing etc.) (Not at all; Hardly at all; A little; A fair amount; A lot; Can't do because of eyesight; Don't do this for other reasons= no score) |
| **IVI 12** | In the past month, how much has your eyesight interfered with opening packaging? (for example, around food, medicines) (Not at all; Hardly at all; A little; A fair amount; A lot; Can't do because of eyesight; Don't do this for other reasons= no score) |
| **IVI 13** | In the past month, how much has your eyesight interfered with reading labels or instructions on medicines? (Not at all; Hardly at all; A little; A fair amount; A lot; Can't do because of eyesight; Don't do this for other reasons= no score) |
| **IVI 14** | In the past month, how much has your eyesight interfered with operating household appliances and the telephone? (Not at all; Hardly at all; A little; A fair amount; A lot; Can't do because of eyesight; Don't do this for other reasons= no score) |
| **IVI 15** | In the past month, how much has your eyesight interfered with reading a sign across the street? (Not at all; Hardly at all; A little; A fair amount; A lot; Can't do because of eyesight; Don't do this for other reasons= no score) |
| **IVI 16** | In the past month, how much has your eyesight interfered with getting about outdoors? (on the pavement or crossing the street) (Not at all; Hardly at all; A little; A fair amount; A lot; Can't do because of eyesight; Don't do this for other reasons= no score) |
| **IVI 17** | In the past month, how often has your eyesight made you go carefully to avoid falling or tripping? (Not at all; Hardly at all; A little; A fair amount; A lot; Can't do because of eyesight; Don't do this for other reasons= no score) |
| **IVI 18** | In general, how much has your eyesight interfered with travelling or using transport? (bus and train) (Not at all; Hardly at all; A little; A fair amount; A lot; Can't do because of eyesight; Don't do this for other reasons= no score) |
| **IVI 19** | In the past month, how much has your eyesight interfered with going down steps, stairs, or curbs? (Not at all; Hardly at all; A little; A fair amount; A lot; Can't do because of eyesight; Don't do this for other reasons= no score) |
| **IVI 20** | In the past month, how much has your eyesight made you concerned or worried about your general safety at home? (Not at all; Very rarely; A little of the time; A fair amount of the time; A lot of the time; All the time) |
| **IVI 21** | In the past month, how much has your eyesight made you concerned or worried about spilling or breaking things? (Not at all; Very rarely; A little of the time; A fair amount of the time; A lot of the time; All the time) |
| **IVI 22** | In the past month, how much has your eyesight made you concerned or worried about your general safety when out of your home? (Not at all; Very rarely; A little of the time; A fair amount of the time; A lot of the time; All the time) |
| **IVI 23** | In the past month, how often has your eyesight stopped you doing the things you want to do? (Not at all; Very rarely; A little of the time; A fair amount of the time; A lot of the time; All the time) |
| **IVI 24** | In the past month, how often have you needed help from other people because of your eyesight? (Not at all; Very rarely; A little of the time; A fair amount of the time; A lot of the time; All the time) |
| **IVI 25** | Think about how your eyesight has made you feel in the past month - have you felt embarrassed because of your eyesight? (Not at all; Very rarely; A little of the time; A fair amount of the time; A lot of the time; All the time) |
| **IVI 26** | Think about how your eyesight has made you feel in the past month - have you felt frustrated or annoyed because of your eyesight? (Not at all; Very rarely; A little of the time; A fair amount of the time; A lot of the time; All the time) |
| **IVI 27** | Think about how your eyesight has made you feel in the past month - have you felt lonely or isolated because of your eyesight? (Not at all; Very rarely; A little of the time; A fair amount of the time; A lot of the time; All the time) |
| **IVI 28** | Think about how your eyesight has made you feel in the past month - have you felt sad or low because of your eyesight? (Not at all; Very rarely; A little of the time; A fair amount of the time; A lot of the time; All the time) |
| **IVI 29** | In the past month, how often have you worried about your eyesight getting worse? (Not at all; Very rarely; A little of the time; A fair amount of the time; A lot of the time; All the time) |
| **IVI 30** | In the past month, how much has your eyesight made you concerned or worried about coping with everyday life? (Not at all; Very rarely; A little of the time; A fair amount of the time; A lot of the time; All the time) |
| **IVI 31** | Think about how your eyesight has made you feel in the past month - have you felt like a nuisance or a burden because of your eyesight? (Not at all; Very rarely; A little of the time; A fair amount of the time; A lot of the time; All the time) |
| **IVI 32** | In the past month, how much has your eyesight interfered with your life in general? (Not at all; Very rarely; A little of the time; A fair amount of the time; A lot of the time; All the time) |

| **16. National Eye Institute Visual Function Questionnaire (NEI-VFQ 25)** | |
| --- | --- |
| **Item** | **Question format (Response categories)** |
| **NEIVFQ 1** | In general, would you say your overall health is (Excellent; Very good; Good; Fair; Poor) |
| **NEIVFQ 2** | At the present time, would you say your eyesight using both eyes (with glasses or contact lenses, if you wear them) is excellent, good, fair, poor, or very poor, or are you completely blind? (Excellent; Good; Fair; Poor; Very Poor; Completely blind) |
| **NEIVFQ 3** | How much of the time do you worry about your eyesight? (None of the time; A little of the time; Some of the time; Most of the time; All of the time) |
| **NEIVFQ 4** | How much pain or discomfort have you had in and around your eyes (for example, burning, itching, or aching)? Would you say it is: (None; Mild; Moderate; Severe; Very severe) |
| **NEIVFQ 5** | How much difficulty do you have reading ordinary print in newspapers? Would you say you have: (No difficulty at all; A little difficulty; Moderate difficulty; Extreme difficulty; Stopped doing this because of your eyesight; Stopped doing this for other reasons = no score) |
| **NEIVFQ 6** | How much difficulty do you have doing work or hobbies that require you to see well up close, such as cooking, sewing, fixing things around the house, or using hand tools? Would you say: (No difficulty at all; A little difficulty; Moderate difficulty; Extreme difficulty; Stopped doing this because of your eyesight; Stopped doing this for other reasons = no score) |
| **NEIVFQ 7** | Because of your eyesight, how much difficulty do you have finding something on a crowded shelf? (No difficulty at all; A little difficulty; Moderate difficulty; Extreme difficulty; Stopped doing this because of your eyesight; Stopped doing this for other reasons = no score) |
| **NEIVFQ 8** | How much difficulty do you have reading street signs or the name of stores? (No difficulty at all; A little difficulty; Moderate difficulty; Extreme difficulty; Stopped doing this because of your eyesight; Stopped doing this for other reasons = no score) |
| **NEIVFQ 9** | Because of your eyesight, how much difficulty do you have going down steps, stairs, or curbs in dim light or at night? (No difficulty at all; A little difficulty; Moderate difficulty; Extreme difficulty; Stopped doing this because of your eyesight; Stopped doing this for other reasons = no score) |
| **NEIVFQ 10** | Because of your eyesight, how much difficulty do you have noticing objects off to the side while you are walking along? (No difficulty at all; A little difficulty; Moderate difficulty; Extreme difficulty; Stopped doing this because of your eyesight; Stopped doing this for other reasons = no score) |
| **NEIVFQ 11** | Because of your eyesight, how much difficulty do you have seeing how people react to things you say? (No difficulty at all; A little difficulty; Moderate difficulty; Extreme difficulty; Stopped doing this because of your eyesight; Stopped doing this for other reasons = no score) |
| **NEIVFQ 12** | Because of your eyesight, how much difficulty do you have picking out and matching your own clothes? (No difficulty at all; A little difficulty; Moderate difficulty; Extreme difficulty; Stopped doing this because of your eyesight; Stopped doing this for other reasons = no score) |
| **NEIVFQ 13** | Because of your eyesight, how much difficulty do you have visiting with people in their own homes, at parties, or in restaurants? (No difficulty at all; A little difficulty; Moderate difficulty; Extreme difficulty; Stopped doing this because of your eyesight; Stopped doing this for other reasons = no score) |
| **NEIVFQ 14** | Because of your eyesight, how much difficulty do you have going out to see movies, plays, or sports events? (No difficulty at all; A little difficulty; Moderate difficulty; Extreme difficulty; Stopped doing this because of your eyesight; Stopped doing this for other reasons = no score) |
| **NEIVFQ 15** | Are you currently driving, at least once in a while? (Yes (skip to Q15c); No) |
| **NEIVFQ 15a** | IF NO: Have you never driven a car or have you given up driving? (Never drove (skip to Part 3,Q17); Gave up) |
| **NEIVFQ 15b** | IF YOU GAVE UP DRIVING: Was that mainly because of your eyesight, mainly for some other reason, or because of both your eyesight and other reasons? (Mainly eyesight (skip to Part 3, Q17); Mainly other reasons (skip to Part 3, Q17) |
| **NEIVFQ 15c** | IF CURRENTLY DRIVING: How much difficulty do you have driving during the daytime in familiar places: Would you say you have: (No difficulty at all; A little difficulty; Moderate difficulty; Extreme difficulty) |
| **NEIVFQ 16** | How much difficulty do you have driving at night? Would you say you have: (No difficulty at all; A little difficulty; Moderate difficulty; Extreme difficulty; Have you stopped doing this because of your eyesight; Have you stopped doing this for other reasons= no score) |
| **NEIVFQ 16a** | How much difficulty do you have driving in difficult conditions, such as in bad weather, during rush hour, on the freeway, or in city traffic? Would you say you have: (No difficulty at all; A little difficulty; Moderate difficulty; Extreme difficulty; Stopped doing this because of your eyesight; Stopped doing this for other reasons = no score) |
| **NEIVFQ 17** | Do you accomplish less than you would like because of your vision? (All of the time; Most of the time; Some of the time; A little of the time; None of the time) |
| **NEIVFQ 18** | Are you limited in how long you can work or do other activities because of your vision? (All of the time; Most of the time; Some of the time; A little of the time; None of the time) |
| **NEIVFQ 19** | How much does pain or discomfort in or around your eyes, for example, burning, itching, or aching, keep you from doing what you'd like to be doing? Would you say: (All of the time; Most of the time; Some of the time; A little of the time; None of the time) |
| **NEIVFQ 20** | I stay home most of the time because of my eyesight. (Definitely true; Mostly true; Not sure; Mostly false; Definitely false) |
| **NEIVFQ 21** | I feel frustrated a lot of the time because of my eyesight. (Definitely true; Mostly true; Not sure; Mostly false; Definitely false) |
| **NEIVFQ 22** | I have much less control over what I do, because of my eyesight. (Definitely true; Mostly true; Not sure; Mostly false; Definitely false) |
| **NEIVFQ 23** | Because of my eyesight, I have to rely too much on what other people tell me. (Definitely true; Mostly true; Not sure; Mostly false; Definitely false) |
| **NEIVFQ 24** | I need a lot of help from others because of my eyesight. (Definitely true; Mostly true; Not sure; Mostly false; Definitely false) |
| **NEIVFQ 25** | I worry about doing things that will embarrass myself or others, because of my eyesight. (Definitely true; Mostly true; Not sure; Mostly false; Definitely false) |
| **NEIVFQ A1** | How would you rate your overall health, on a scale where zero is as bad as death and 10 is best possible health? (Linear scale from Worst=0 to Best=10) |
| **NEIVFQ A2** | How would you rate your eyesight now (with glasses or contact lens on, if you wear them), on a scale of from 0 to 10, where zero means the worst possible eyesight, as bad or worse than being blind, and 10 means the best possible eyesight? (Linear scale numerical; 0-10) |
| **NEIVFQ A3** | Wearing glasses, how much difficulty do you have reading the small print in a telephone book, on a medicine bottle, or on legal forms? Would you say: (No difficulty at all; A little difficulty; Moderate difficulty; Extreme difficulty; Stopped doing this because of your eyesight; Stopped doing this for other reasons =no score) |
| **NEIVFQ A4** | Because of your eyesight, how much difficulty do you have figuring out whether bills you receive are accurate? (No difficulty at all; A little difficulty; Moderate difficulty; Extreme difficulty; Stopped doing this because of your eyesight; Stopped doing this for other reasons =no score) |
| **NEIVFQ A5** | Because of your eyesight, how much difficulty do you have doing things like shaving, styling your hair, or putting on makeup? (No difficulty at all; A little difficulty; Moderate difficulty; Extreme difficulty; Stopped doing this because of your eyesight; Stopped doing this for other reasons =no score) |
| **NEIVFQ A6** | Because of your eyesight, how much difficulty do you have recognising people you know from across a room? (No difficulty at all; A little difficulty; Moderate difficulty; Extreme difficulty; Stopped doing this because of your eyesight; Stopped doing this for other reasons =no score) |
| **NEIVFQ A7** | Because of your eyesight, how much difficulty do you have taking part in active sports or other outdoor activities that you enjoy (like golf, bowling, jogging, or walking)? (No difficulty at all; A little difficulty; Moderate difficulty; Extreme difficulty; Stopped doing this because of your eyesight; Stopped doing this for other reasons =no score) |
| **NEIVFQ A8** | Because of your eyesight, how much difficulty do you have seeing and enjoying programs on TV? (No difficulty at all; A little difficulty; Moderate difficulty; Extreme difficulty; Stopped doing this because of your eyesight; Stopped doing this for other reasons =no score) |
| **NEIVFQ A9** | Because of your eyesight, how much difficulty do you have entertaining friends and family in your home? (No difficulty at all; A little difficulty; Moderate difficulty; Extreme difficulty; Stopped doing this because of your eyesight; Stopped doing this for other reasons =no score) |
| **NEIVFQ A11a** | Do you have more help from others because of your vision? (All of the time; Most of the time; Some of the time; A little of the time; None of the time) |
| **NEIVFQ A11b** | Are you limited in the kinds of things you can do because of your vision? (All of the time; Most of the time; Some of the time; A little of the time; None of the time) |
| **NEIVFQ A12** | I am often irritable because of my eyesight (Definitely true; Mostly true; Not sure; Most false; Definitely false) |
| **NEIVFQ A13** | I don't go out of my home alone, because of my eyesight (Definitely true; Mostly true; Not sure; Most false; Definitely false) |

| **17. Visual Symptoms and Quality of Life Questionnaire (VSQ)** | |
| --- | --- |
| **Item** | **Question format (Response categories)** |
| **VSQ 1** | Do you have difficulty reading normal print in books or newspapers because of trouble with your eyesight? (No difficulty; Yes, a little difficulty; Yes, some difficulty; Yes, a great deal of difficulty; I cannot read because of my eyesight) |
| **VSQ 2** | Do you have difficulty recognising people's faces because of trouble with your eyesight? (No difficulty; Yes, but only at a distance, for example across a main street; Yes, even at close range; I cannot see faces at all now) |
| **VSQ 3** | When you are watching television, do you find it difficult to see the picture clearly? (No, not difficult ; Yes, a little difficult ; Yes, quite difficult ; Yes, very difficult ; I cannot see the picture on the television at all ; I do not watch television because of my eyesight) |
| **VSQ 4** | Do you feel that your bad eye is affecting or interfering with your vision? (No, never ; Yes, some of the time ; Yes, most of the time; Yes, all of the time) |
| **VSQ 4b** | Which is your bad eye? (Right; Left) |
| **VSQ 5** | Is it difficult for you to read signs in the streets, shops or other public places? (No, not difficult; Yes, a little difficult; Yes, quite difficult; Ye, very difficult; I cannot see signs now because of my eyesight) |
| **VSQ 6** | Do you have difficulty judging steps or kerbs because of trouble with your eyesight? (No difficulty ; Yes, a little difficulty; Yes, some difficulty; Yes, a great deal of difficulty; I cannot judge steps or kerbs now because of my eyesight) |
| **VSQ 7** | Do you find it difficult to judge distances, for example how far away cars are when you want to cross the road? (No, not difficult : Yes, a little difficult; Yes, quite difficult; Yes, very difficult; I cannot judge distances because of trouble with my eyesight) |
| **VSQ 8** | How much difficulty do you have pouring liquids (water, tea, coffee from a jug or pot into a cup) because of trouble with your eyesight? (No difficulty; Yes, a little difficulty; Yes, some difficulty; Yes, a great deal of difficulty; I cannot pour the liquid) |
| **VSQ 9** | Do you find it difficult to see in bright light, such a strong sunshine or fluorescent / spot lights? (No difficulty; Yes, a little difficulty; Yes, some difficulty; Yes, a great deal of difficulty; I cannot see at all in bright light ) |
| **VSQ 10** | Do you shut or cover one eye so that you can see better? (No, never ; Yes, some of the time; Yes, most of the time; Yes, all of the time ) |
| **VSQ 11** | During the past month, have you had misty or foggy vision? (No, never; Yes, some of the time; Yes, most of the time; Yes, all of the time) |
| **VSQ 12** | Are you dazzled by bright light, such as strong sunshine, fluorescent lights or car headlights? (No, never; Yes, some of the time; Yes, most of the time; Yes, all of the time) |
| **VSQ 13** | Do you have blurry vision? (No, never; Yes, some of the time; Yes, most of the time; Yes, all of the time) |
| **VSQ 14** | Do you screw up your eyes so that you can see better? (No, never; Yes, some of the time; Yes, most of the time; Yes, all of the time) |
| **VSQ 15 (1)** | How often does you eyesight prevent you from doing the things you would like to do? (Never; Some of the time; Most of the time; All of the time) |
| **VSQ 16 (2)** | Have you ever had to give up paid work because of trouble with your eyesight? (No, I have not had to give up work; I have not had to give up work but I am having difficulty with my job because of my eyesight; I retired/ gave up work because of trouble with your eyesight) |
| **VSQ 17 (3)** | In the past month, have you had any accidents inside the house (for example tripping or falling over things, bumping into things, burning yourself on the cooker, or burning food) because of trouble with your sight? (No; Yes) |
| **VSQ 18(4)** | During the past month, have you found it difficult to do everyday activities around the house or garden, (such as keeping the house clean, mending things, planting), because you cannot see well enough? (No, not difficult; Yes, a little difficult; Yes, quite difficult; Yes, very difficult; I cannot see the picture on the television at all; I do not the activity) |
| **VSQ 19 (5)** | Do you worry that you might be involved in an accident outside the house (falling down steps, bumping into objects) because of your eyesight? (No, never; Yes, some of the time; Yes, most of the time; Yes, all of the time; I cannot go outside) |
| **VSQ 20 (6)** | How would you feel if you had to spend the rest of your life with your eyesight as it is now? (Perfectly happy; Pleased: Mostly satisfied: Mixed feelings; Mostly dissatisfied; Very unhappy; Desperate) |
| **VSQ 21 (7)** | Thinking about your eyesight as it is now, are you able to do all the activities, hobbies or past-times you would like to do? (Yes, I can do all the activities, hobbies or pastimes I would like to do; No there are some activities, hobbies or pastimes) |
| **VSQ 22(8)** | Overall, how much do problems with your eyesight interfere with your life? (not at all ; a little; quite a lo; a great deal) |
| **VSQ 23(9)** | Have you had to give up driving because of trouble with your eyesight? (No, I still drive ; No, I have given up driving for some other reason ; Yes, I have given up driving because of my eyesight) |
| **VSQ V1** | How would you describe your vision overall - with both eyes open, wearing glasses and contact lenses if you usually do? (excellent; very good ; quite good ; average ; quite poor ; very poor; appalling ) |
| **VSQ V2** | During the past month, how would you describe the vision in your left eye with your right eye closed? (excellent; very good ; quite good ; average ; quite poor ; very poor; appalling; cannot see at all ) |
| **VSQ V3** | During the past month, how would you describe the vision in your right eye with your right eye closed? (excellent; very good ; quite good ; average ; quite poor ; very poor; appalling; cannot see at all ) |
